# Supplementary material for: New e:b-Friedo-Hopane Type Triterpenoids from Euphorbia peplus with Simiarendiol Possessing Significant Cytostatic Activity against HeLa Cells by Induction of Apoptosis and S/G2 Cell Cycle Arrest
Source: Molecules. 2019 Aug 27;24(17):3106. doi: 10.3390/molecules24173106 (PMC6749284; doi:10.3390/molecules24173106)
Supplement: Supplementary file 1 [file molecules-24-03106-s001.pdf]

Supplementary data for

New E:B-*friedo*-Hopane Type Triterpenoids from *Euphorbia peplus* with Simiarendiol Possessing Significant Cytostatic Activity against HeLa Cells by Induction of Apoptosis and S/G2 Cell Cycle Arrest

Jin-Hai Yu, Dong-Xiang Wu, Zhi-Pu Yu, Yu-Peng Li, Yin-Yin Wang, Shu-Juan Yu and Hua Zhang*

School of Biological Science and Technology, University of Jinan, 336 West Road of Nan Xinzhuang, Jinan 250022, China

* Corresponding author. E-mail: bio_zhangh@ujn.edu.cn

Table of Contents

Table S1. The preliminary cytotoxic screening results for compounds **1−12** at 30 μM 1
Table S2. Re-optimized conformers, energies and proportions for **1** 2
Table S3. Re-optimized conformers, energies and proportions for **3** 3
Table S4. Re-optimized conformers, energies and proportions for **5** 4
Figure S1. ^{1}H NMR spectrum of **1** in $CDCl_3$ 5
Figure S2. ^{13}C NMR spectrum of **1** in $CDCl_3$ 6
Figure S3. ^{1}H−^{1}H COSY NMR spectrum of **1** in $CDCl_3$ 7
Figure S4. HSQC NMR spectrum of **1** in $CDCl_3$...8
Figure S5. HMBC NMR spectrum of **1** in $CDCl_3$...9
Figure S6. ROESY NMR spectrum of **1** in $CDCl_3$..10
Figure S7. (+)-HRESIMS spectrum of **1** 11
Figure S8. ^{1}H NMR spectrum of **2** in $CDCl_3$ 12
Figure S9. ^{13}C NMR spectrum of **2** in $CDCl_3$ 13
Figure S10. ^{1}H−^{1}H COSY NMR spectrum of **2** in $CDCl_3$ 14
Figure S11. HSQC NMR spectrum of **2** in $CDCl_3$...15
Figure S12. HMBC NMR spectrum of **2** in $CDCl_3$...16
Figure S13. ROESY NMR spectrum of **2** in $CDCl_3$..17
Figure S14. (+)-LRESIMS spectrum of **2** 18
Figure S15. (+)-HRESIMS spectrum of **2**...19
Figure S16. ^{1}H NMR spectrum of **3** in $CDCl_3$ 20
Figure S17. ^{13}C NMR spectrum of **3** in $CDCl_3$ 21
Figure S18. ^{1}H−^{1}H COSY NMR spectrum of **3** in $CDCl_3$ 22
Figure S19. HSQC NMR spectrum of **3** in $CDCl_3$...23
Figure S20. HMBC NMR spectrum of **3** in $CDCl_3$...24
Figure S21. ROESY NMR spectrum of **3** in $CDCl_3$..25
Figure S22. (+)-LRESIMS spectrum of **3** 26
Figure S23. (+)-HRESIMS spectrum of **3**...27
Figure S24. UV spectrum of **3**..28
Figure S25. ^{1}H NMR spectrum of **4** in $CDCl_3$ 29
Figure S26. ^{13}C NMR spectrum of **4** in $CDCl_3$ 30
Figure S27. ^{1}H−^{1}H COSY NMR spectrum of **4** in $CDCl_3$ 31
Figure S28. HSQC NMR spectrum of **4** in $CDCl_3$...32
Figure S29. HMBC NMR spectrum of **4** in $CDCl_3$...33
Figure S30. ROESY NMR spectrum of **4** in $CDCl_3$..34
Figure S31. (+)-LRESIMS spectrum of **4** 35
Figure S32. (+)-HRESIMS spectrum of **4**...36
Figure S33. UV spectrum of **4**..37
Figure S34. ^{1}H NMR spectrum of **5** in C_5D_5N..38
Figure S35. ^{13}C NMR spectrum of **5** in C_5D_5N..39
Figure S36. ^{1}H−^{1}H COSY NMR spectrum of **5** in C_5D_5N...40
Figure S37. HSQC NMR spectrum of **5** in C_5D_5N 41
Figure S38. HMBC NMR spectrum of **5** in C_5D_5N 42

Figure S39. ROESY NMR spectrum of **5** in C_5D_5N 43
Figure S40. (+)-LRESIMS spectrum of **5** 44
Figure S41. (+)-HRESIMS spectrum of **5**..45
Figure S42. UV spectrum of **5**...46
Figure S43. The HPLC analysis for the purity of compound **1** 47
Figure S44. The HPLC analysis for the purity of compound **2** 48
Figure S45. The HPLC analysis for the purity of compound **3** 49
Figure S46. The HPLC analysis for the purity of compound **4** 50
Figure S47. The HPLC analysis for the purity of compound **5** 51

Table S1. The preliminary cytotoxic screening results for compounds **1**–**12** at 30 μM

Compds.	HeLa	A549	MCF-7	MDA-MB-231
**1**	46.98%	40.23%	22.60%	46.01%
**2**	96.82%	98.52%	94.44%	80.22%
**3**	-6.82%	6.32%	-4.61%	-3.09%
**4**	3.78%	12.12%	23.34%	29.44%
**5**	27.22%	23.36%	-1.74%	23.57%
**6**	42.41%	-16.16%	1.36%	2.85%
**7**	47.25%	17.07%	3.68%	10.63%
**8**	15.77%	28.09%	13.56%	21.63%
**9**	-12.59%	10.32%	1.45%	20.21%
**10**	9.63%	-15.30%	10.94%	5.52%
**11**	44.02%	12.36%	23.41%	47.55%
**12**	-0.24%	-3.75%	0.38%	19.12%

Table S2. Re-optimized conformers, energies and proportions for **1**

Number	Conformer	Energy (hartree)	Energy (kcal/mol)	Proportion (%)
1	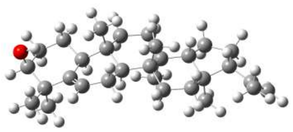	-1247.5965011	-782879.280405261	39.47
2	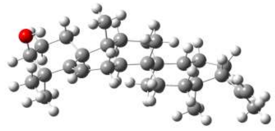	-1247.5952798	-782878.514027298	10.81
3	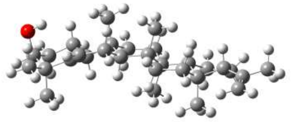	-1247.5930675	-782877.125786925	1.04
4	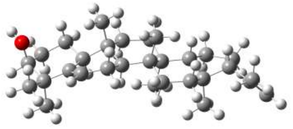	-1247.5956592	-782878.752104592	16.17
5	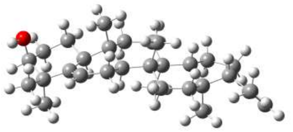	-1247.5959064	-782878.907225064	21.01
6	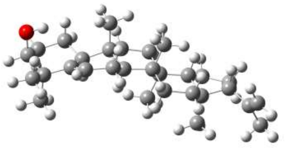	-1247.5953387	-782878.550987637	11.51

Table S3. Re-optimized conformers, energies and proportions for **3**

Number	Conformer	Energy (hartree)	Energy (kcal/mol)	Proportion (%)
1	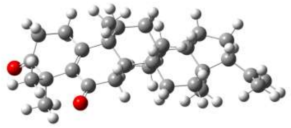	-1320.4351139	-828586.238323389	67.93
2	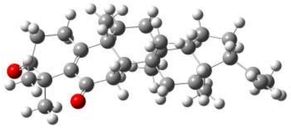	-1320.4340493	-828585.570276243	21.97
3	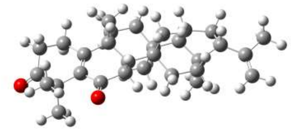	-1320.4316955	-828584.093243205	1.81
4	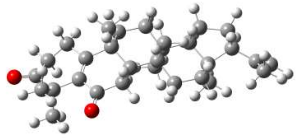	-1320.4331291	-828584.992841541	8.28

Table S4. Re-optimized conformers, energies and proportions for **5**

Number	Conformer	Energy (hartree)	Energy (Kcal/mol)	Proportion (%)
1	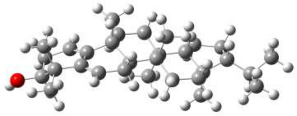	-1247.6113009	-782888.567427759	23.85
2	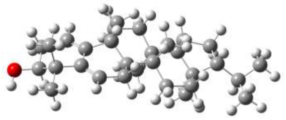	-1247.6118684	-782888.923539684	43.53
3	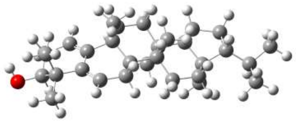	-1247.6115965	-782888.752919715	32.63

Figure S1. ^{1}H NMR spectrum of **1** in $CDCl_3$

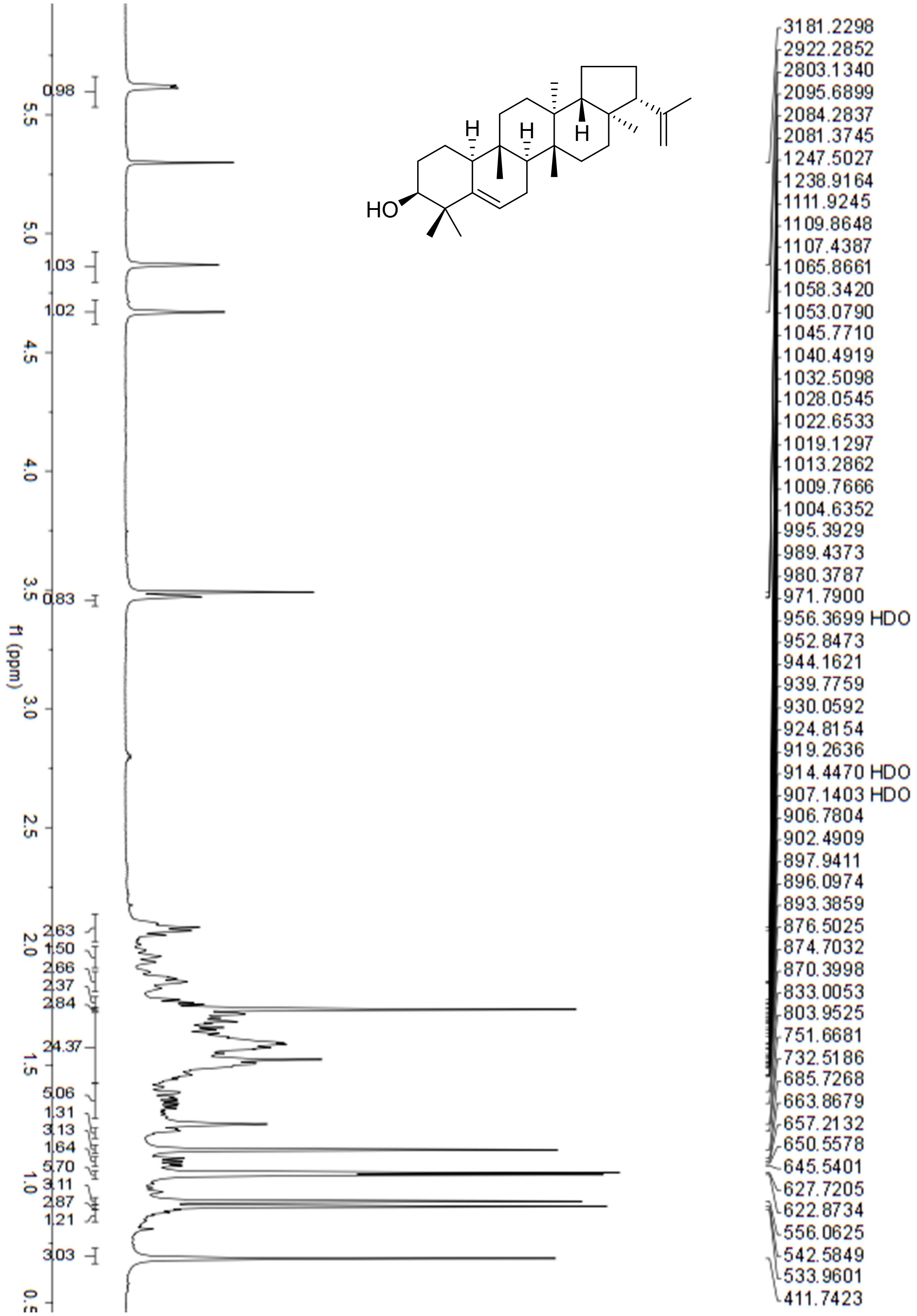

Figure S2. ^{13}C NMR spectrum of **1** in $CDCl_3$

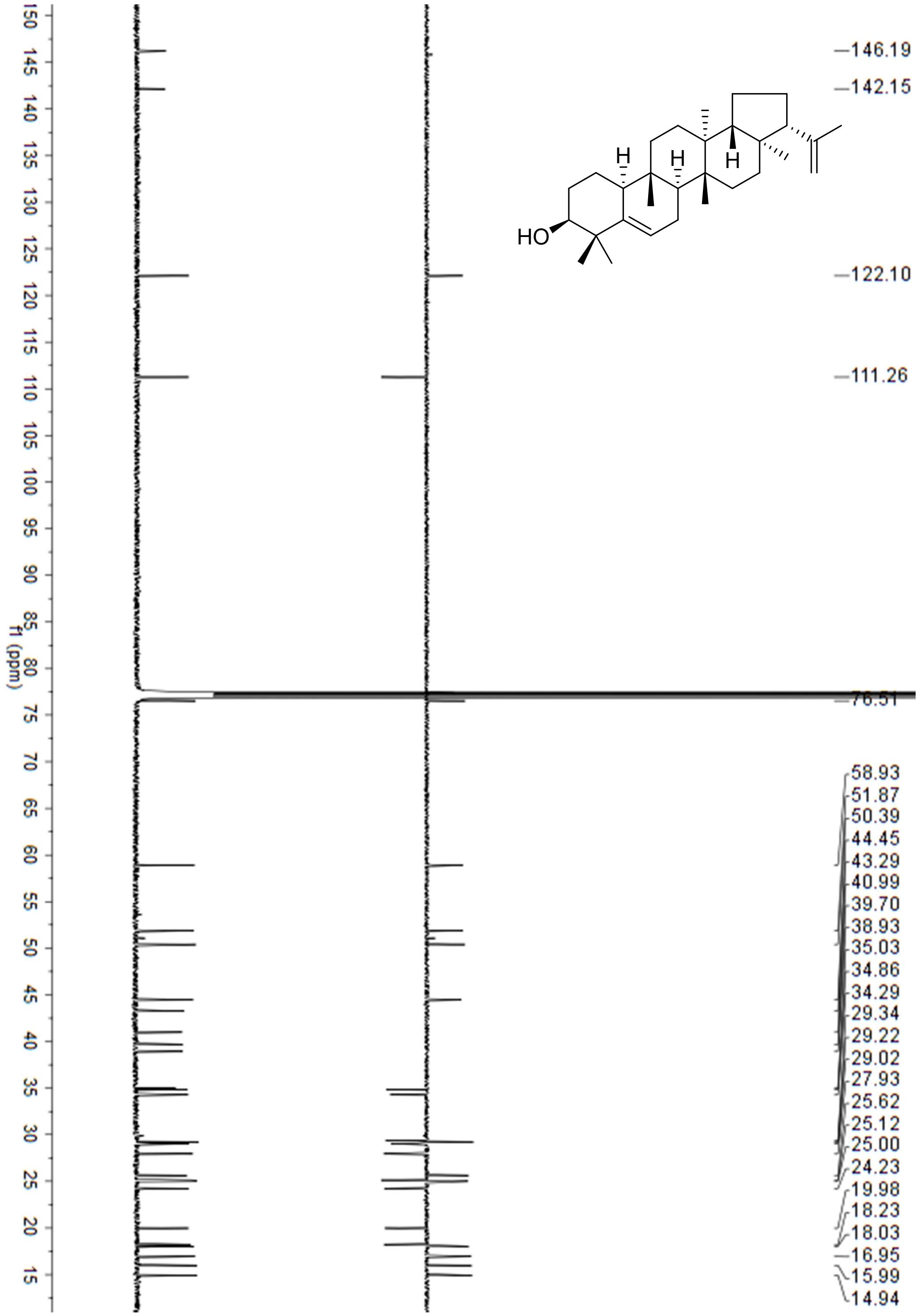

Figure S3. ^{1}H–^{1}H COSY NMR spectrum of **1** in $CDCl_3$

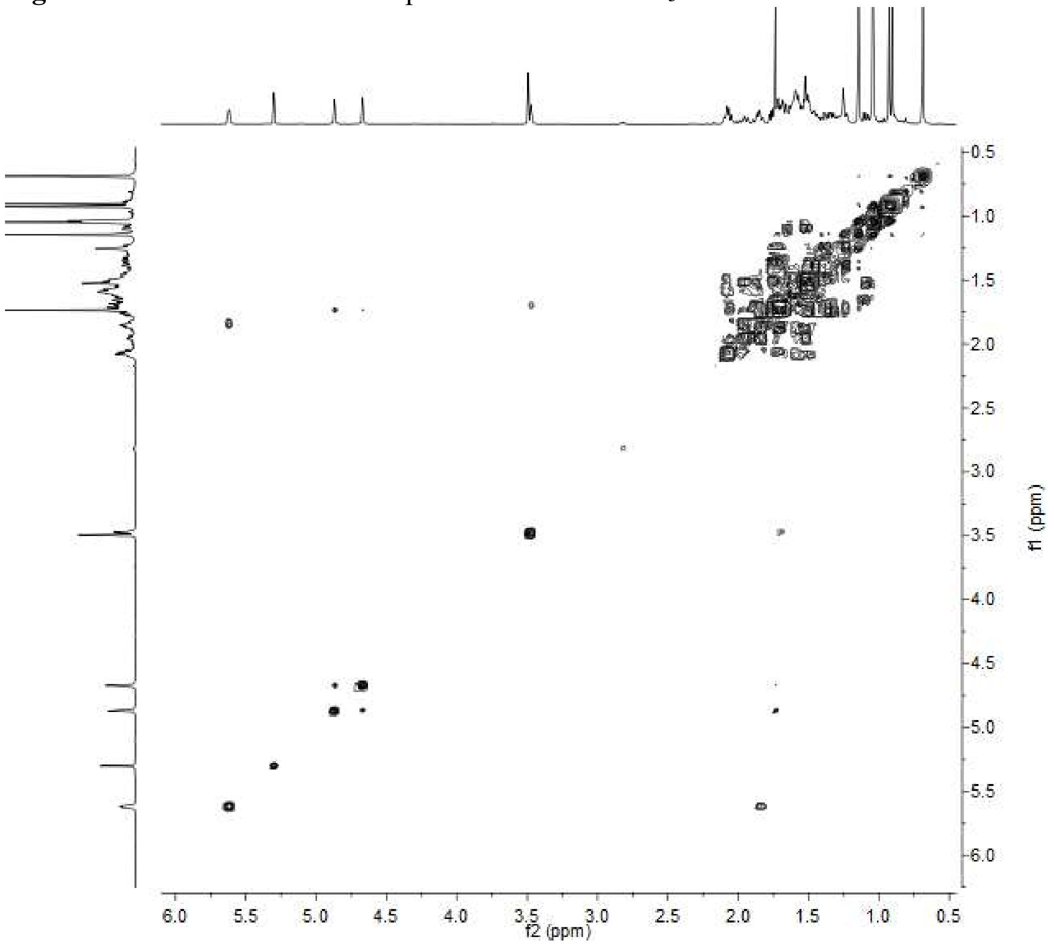

Figure S4. HSQC NMR spectrum of **1** in $CDCl_3$

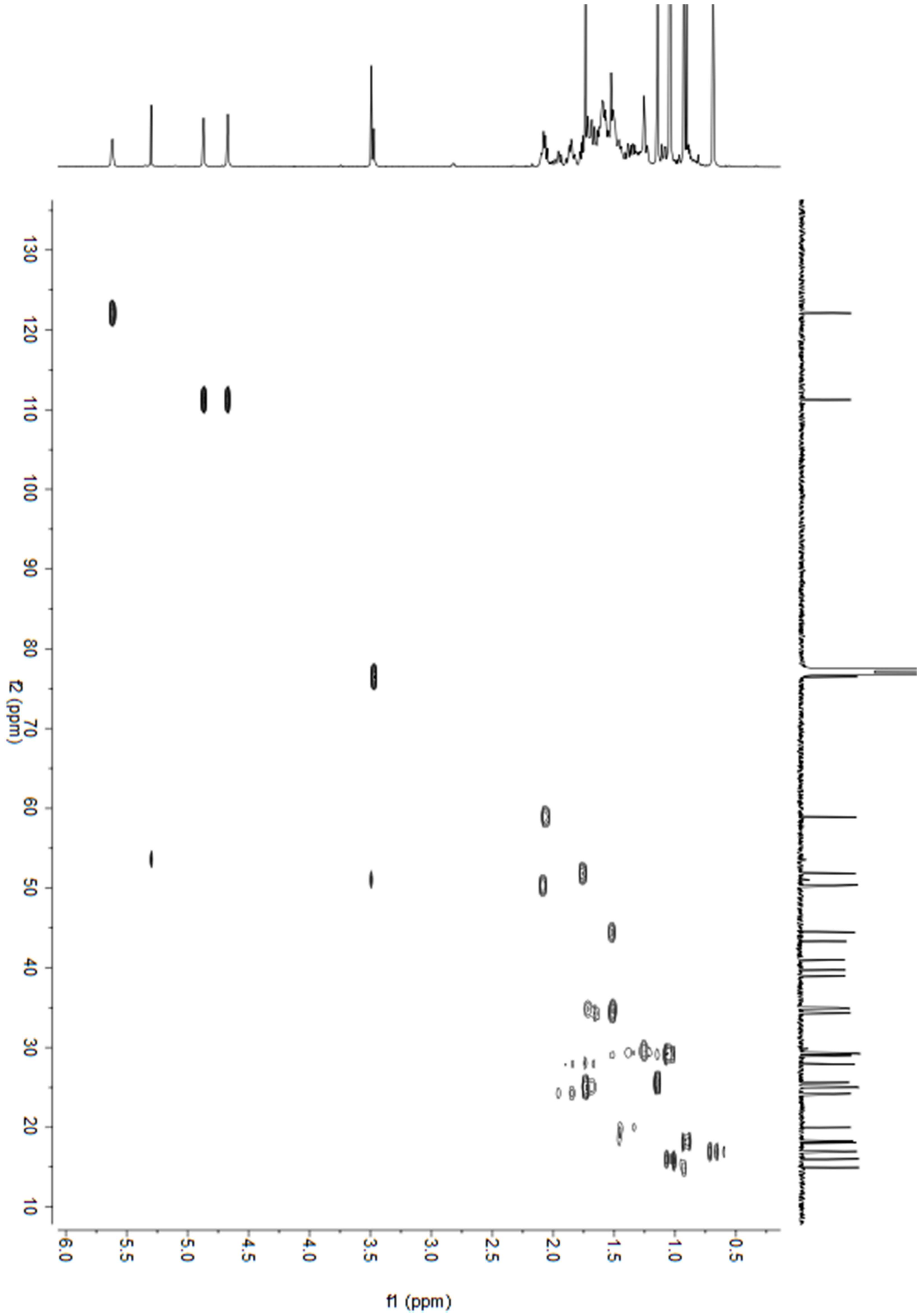

Figure S5. HMBC NMR spectrum of **1** in $CDCl_3$

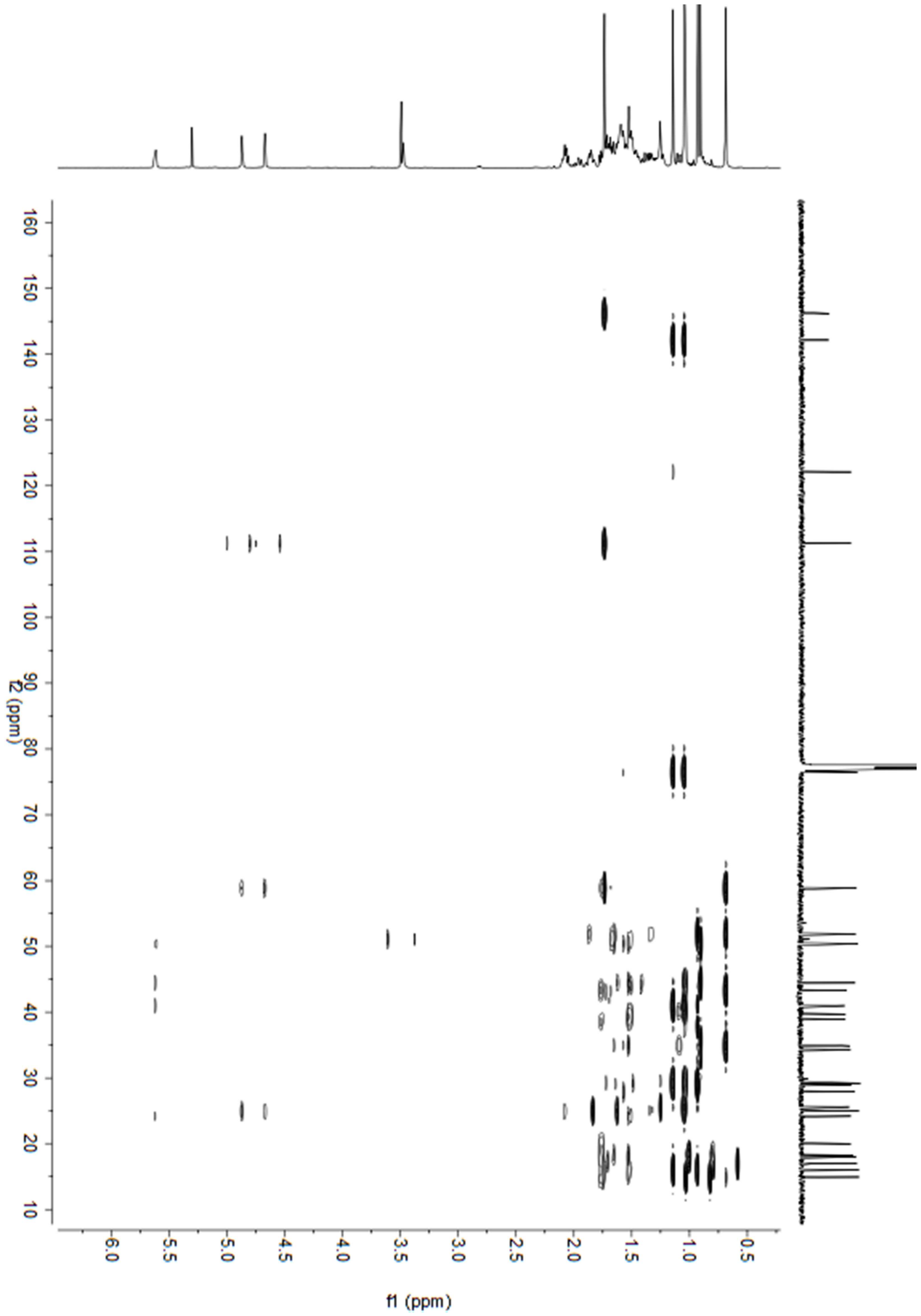

Figure S6. ROESY NMR spectrum of **1** in $CDCl_3$

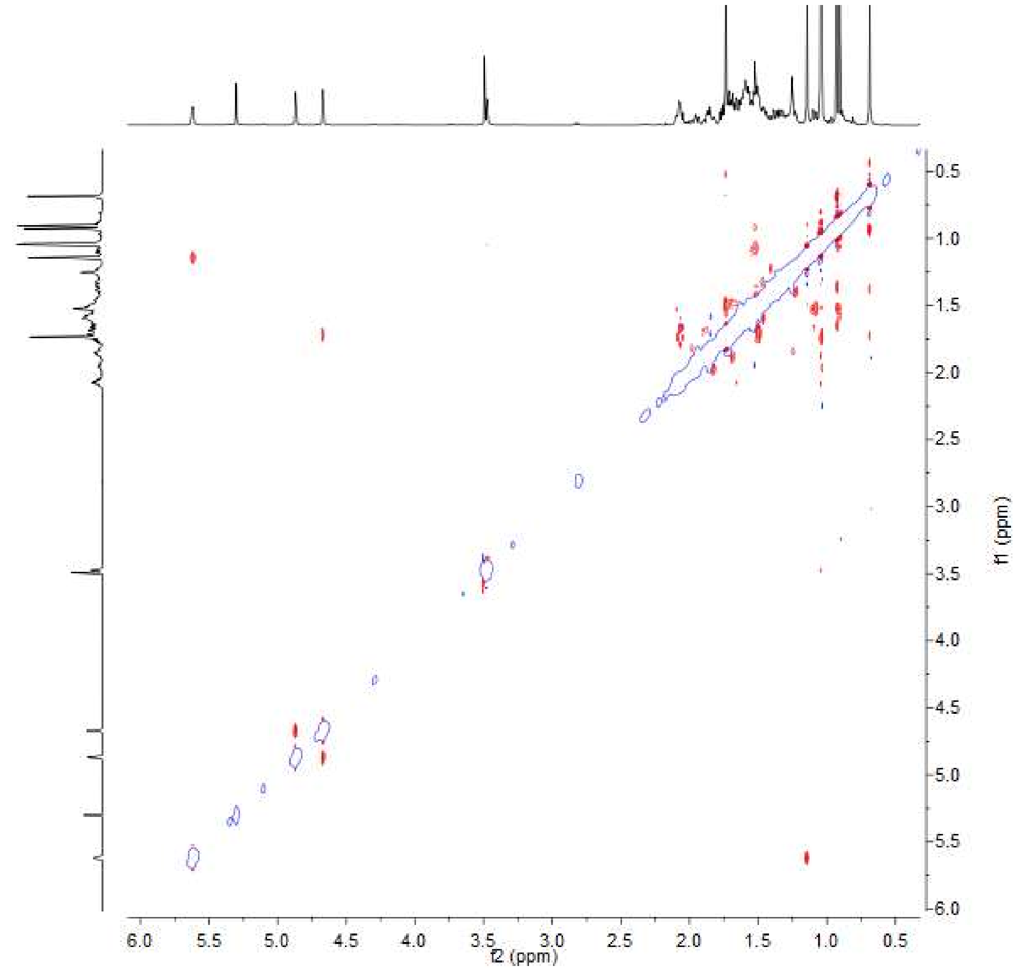

Figure S7. (+)-HRESIMS spectrum of **1**

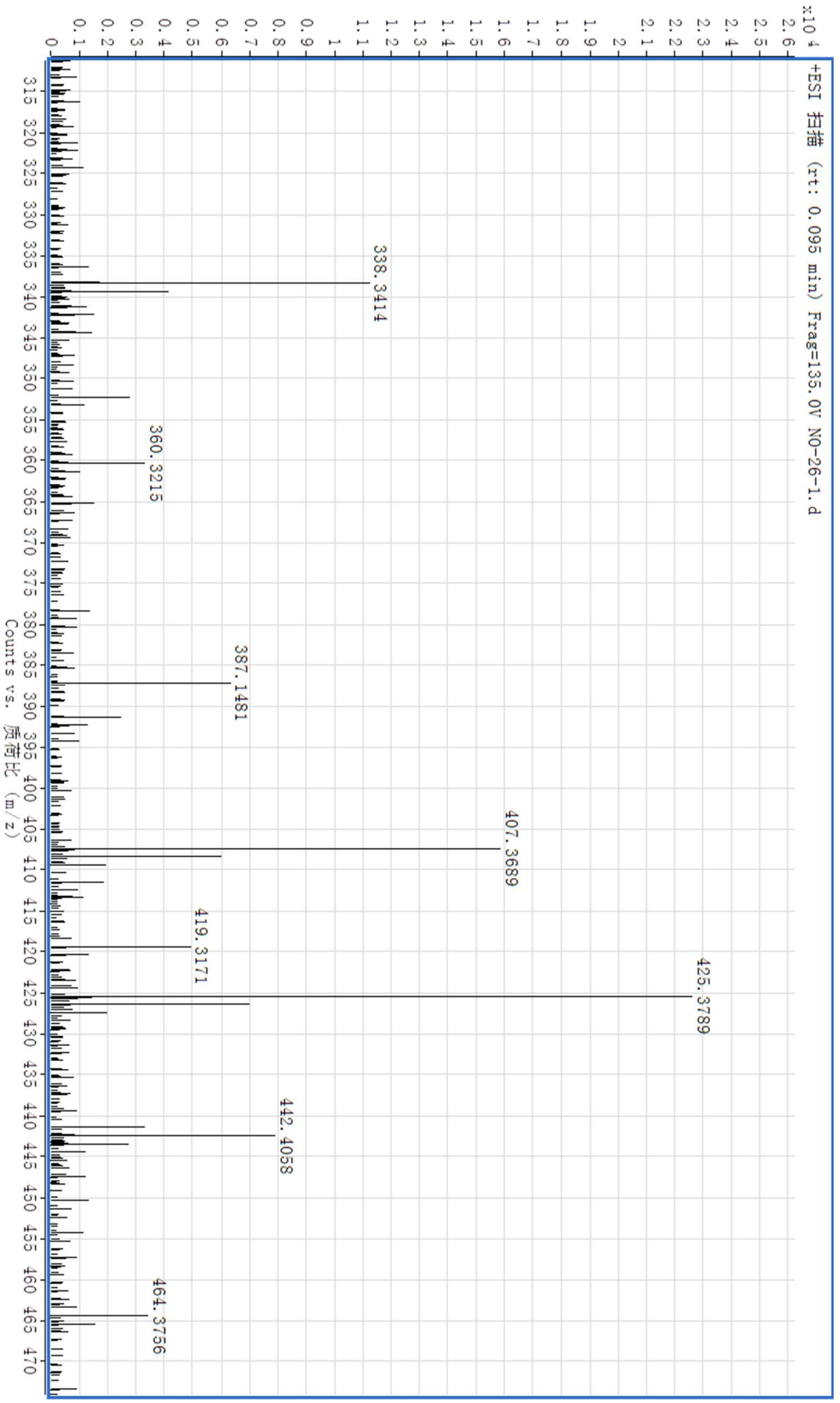

Figure S8. ^{1}H NMR spectrum of **2** in $CDCl_3$

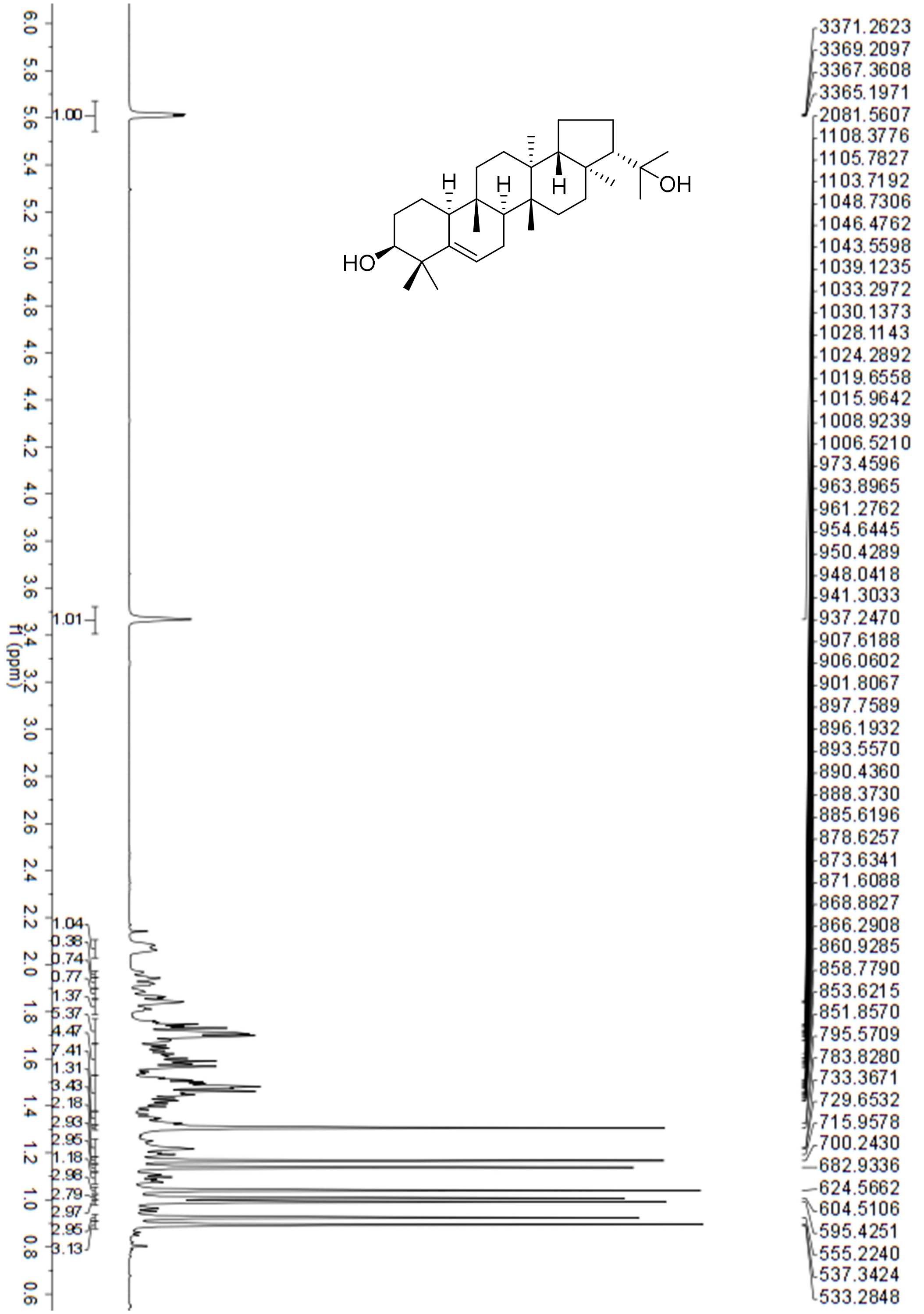

Figure S9. ^{13}C NMR spectrum of **2** in $CDCl_3$

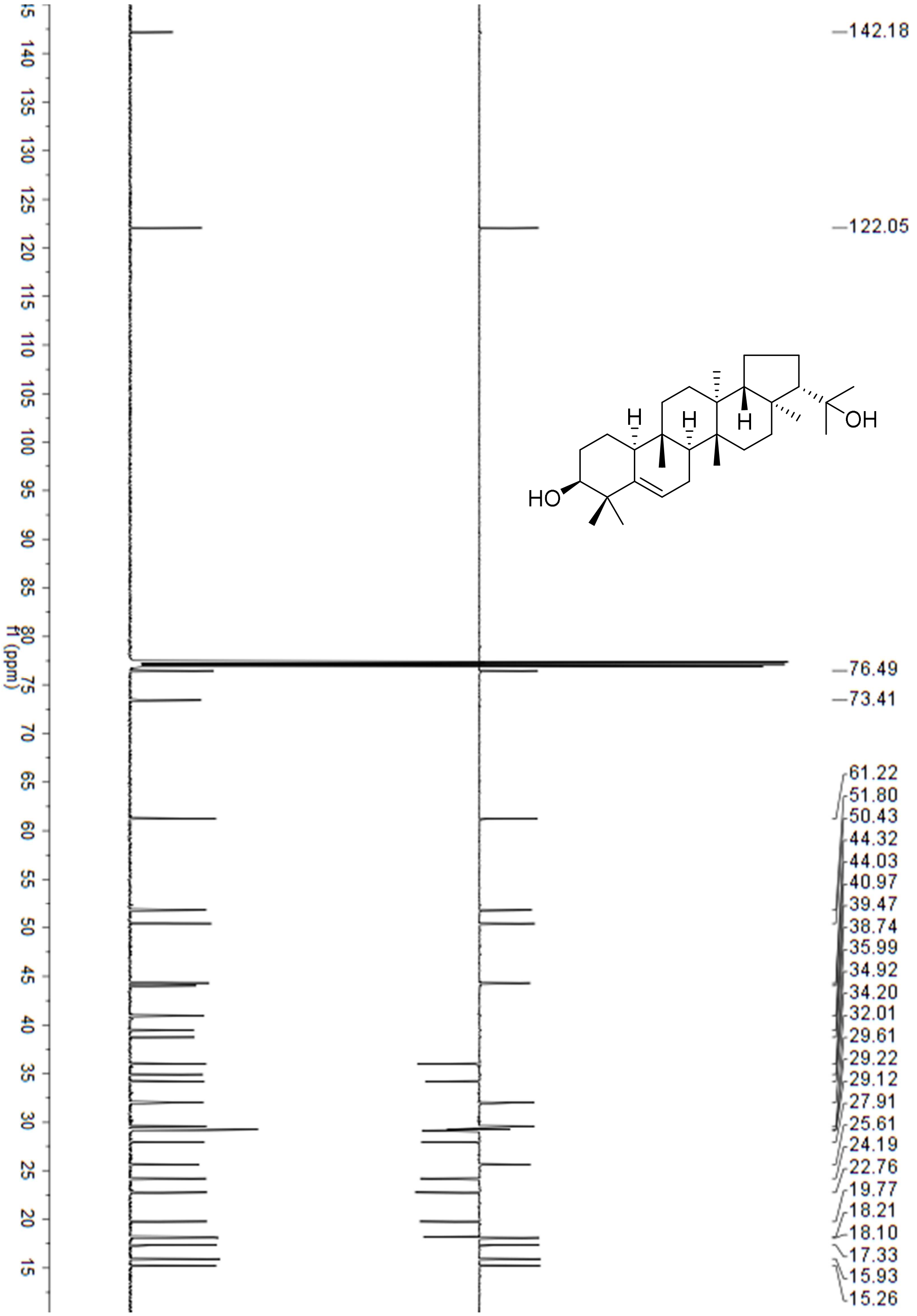

Figure S10. ^{1}H–^{1}H COSY NMR spectrum of **2** in $CDCl_3$

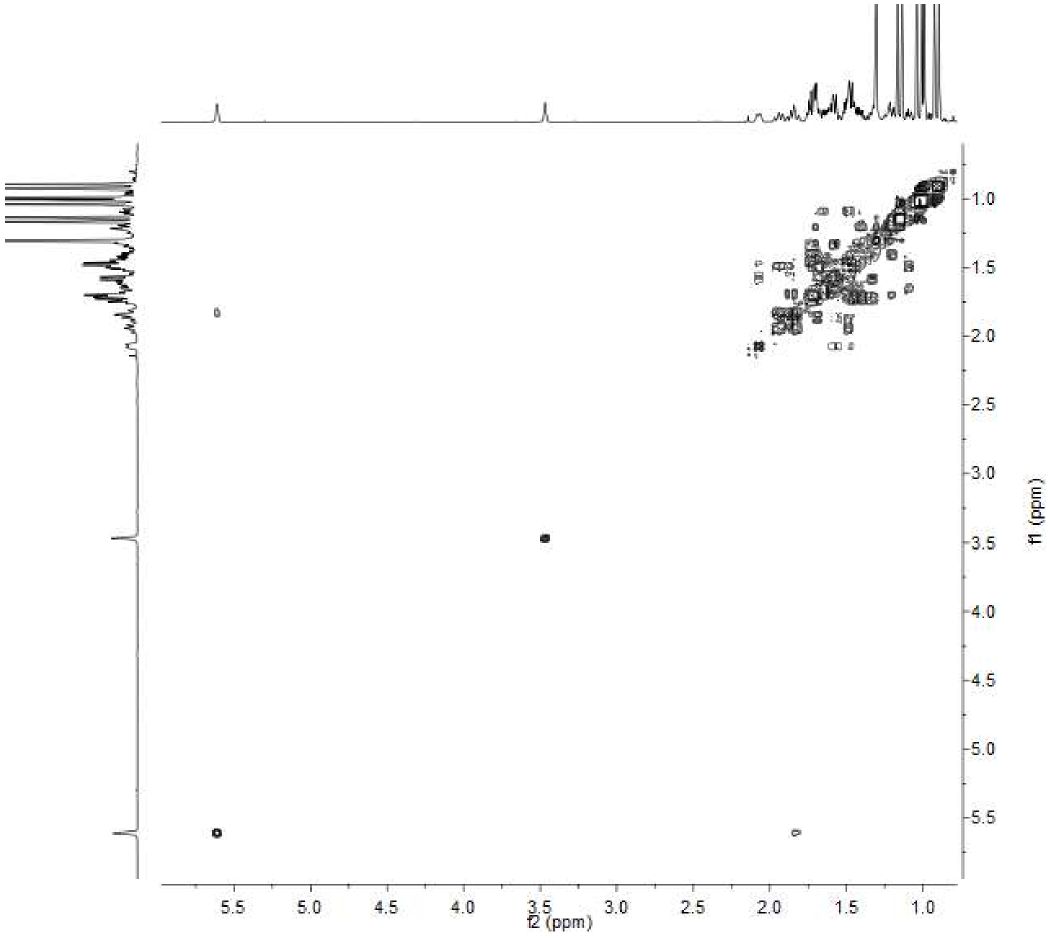

Figure S11. HSQC NMR spectrum of **2** in $CDCl_3$

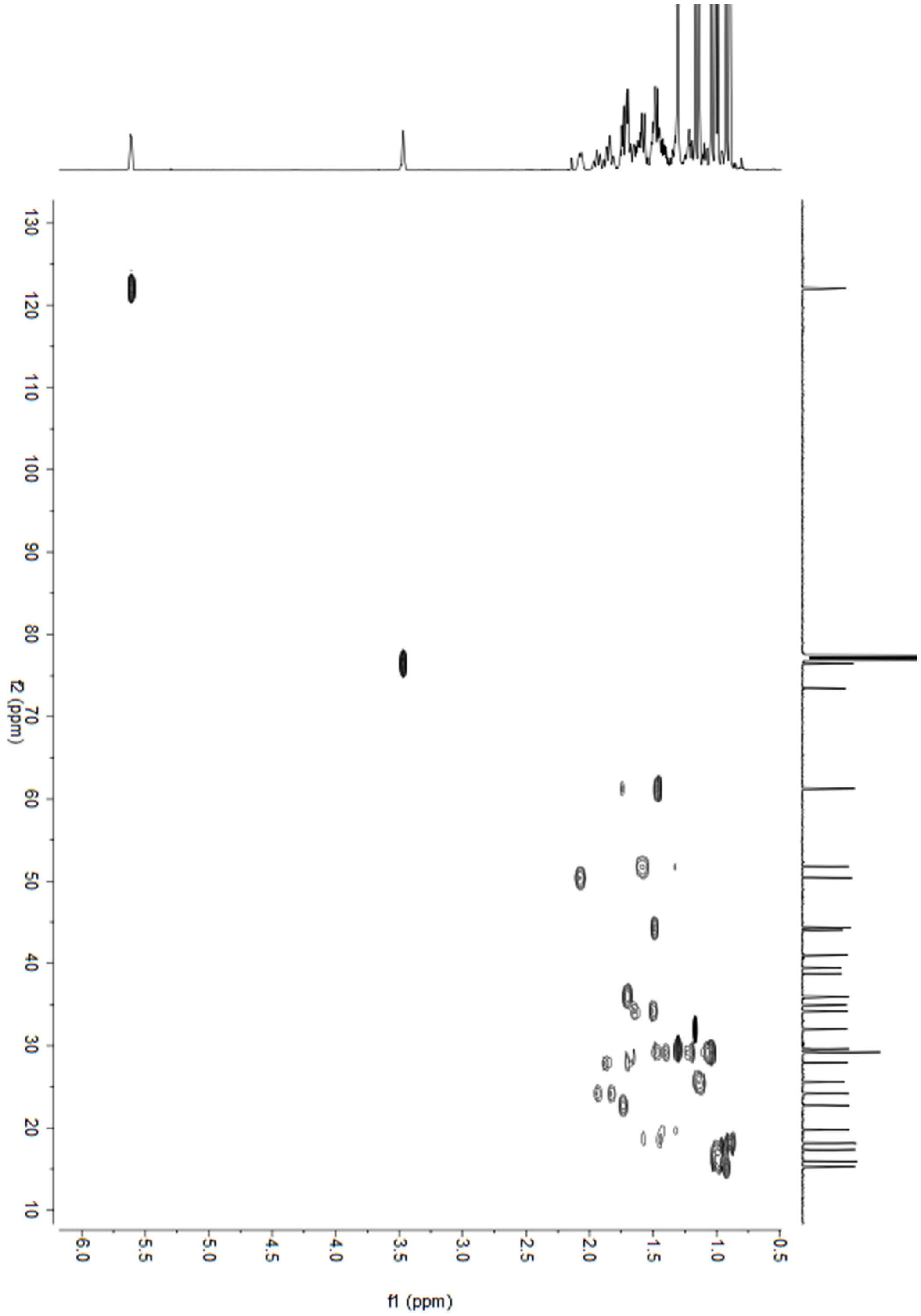

Figure S12. HMBC NMR spectrum of **2** in $CDCl_3$

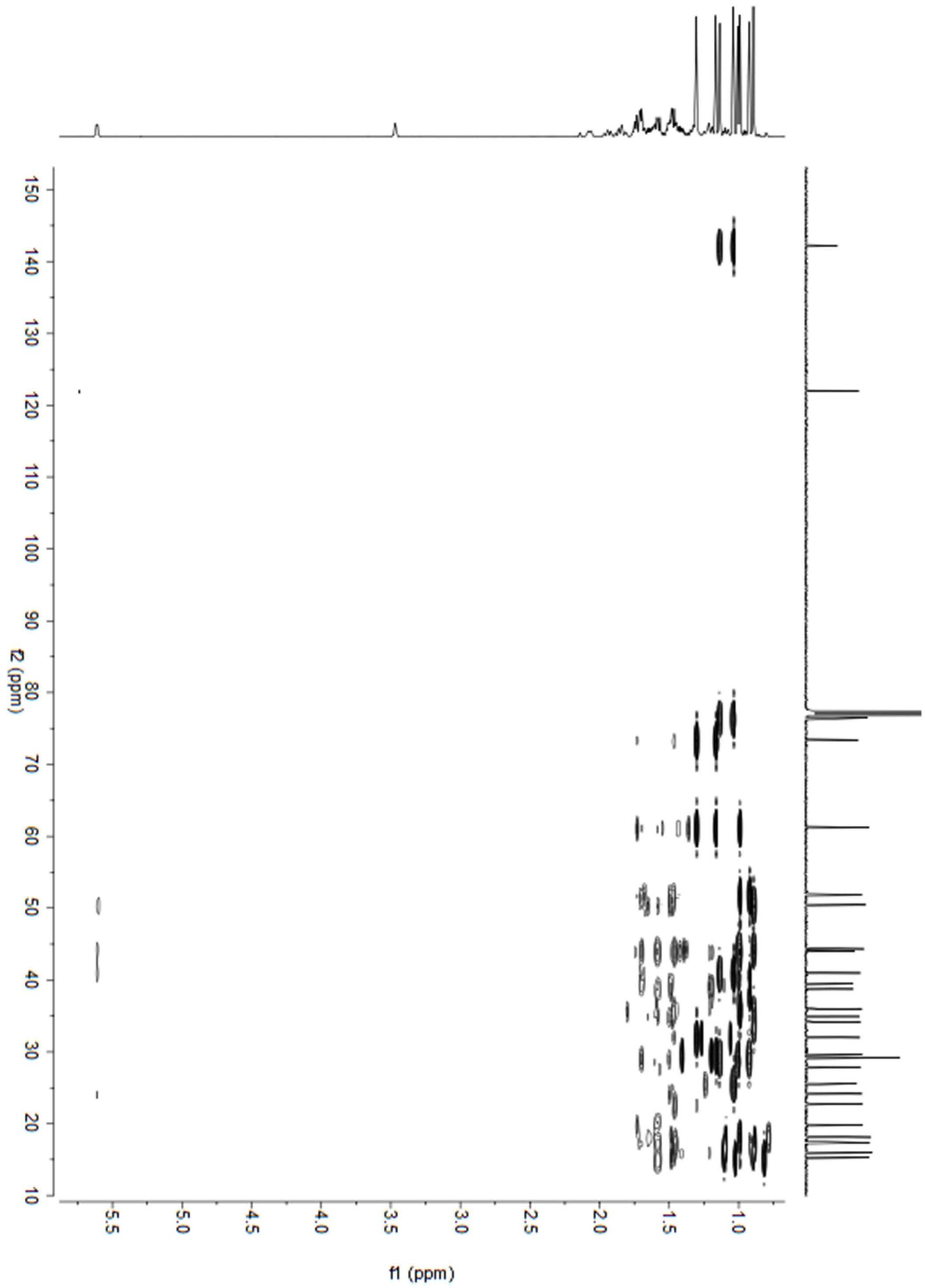

Figure S13. ROESY NMR spectrum of **2** in $CDCl_3$

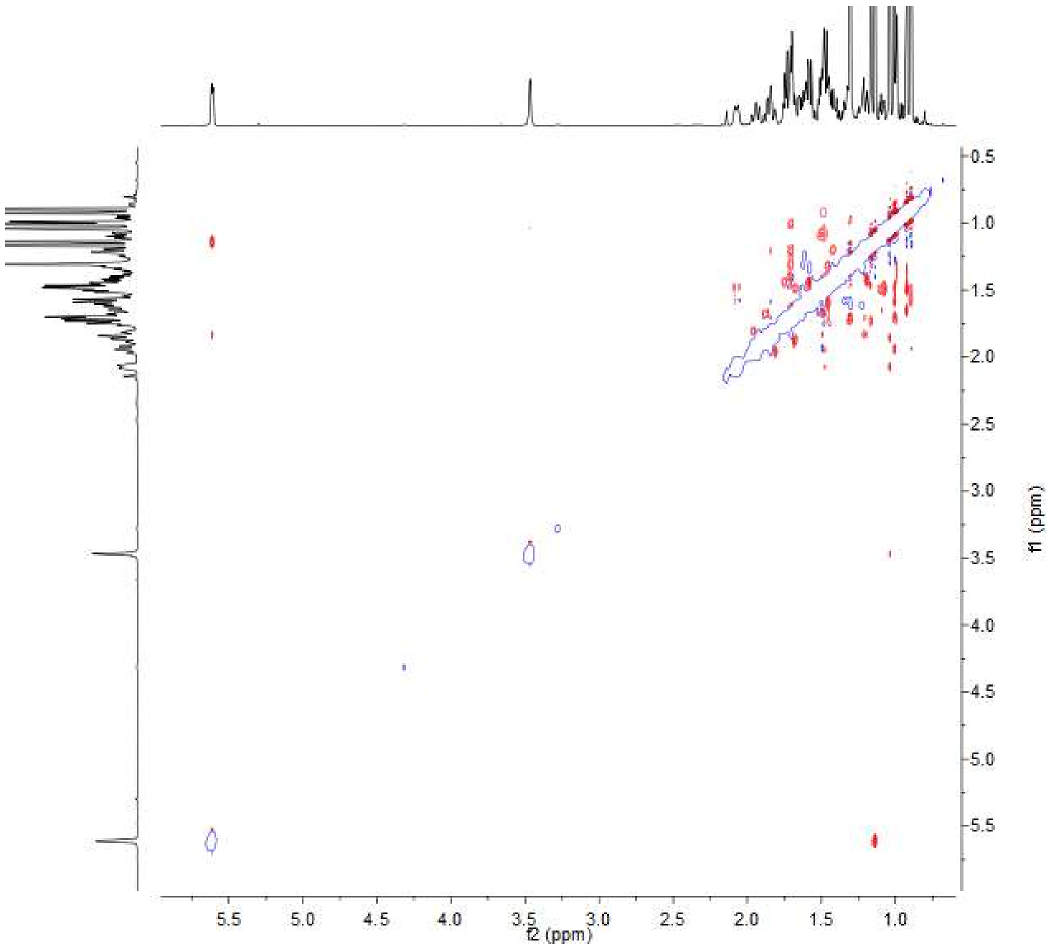

Figure S14. (+)-LRESIMS spectrum of **2**

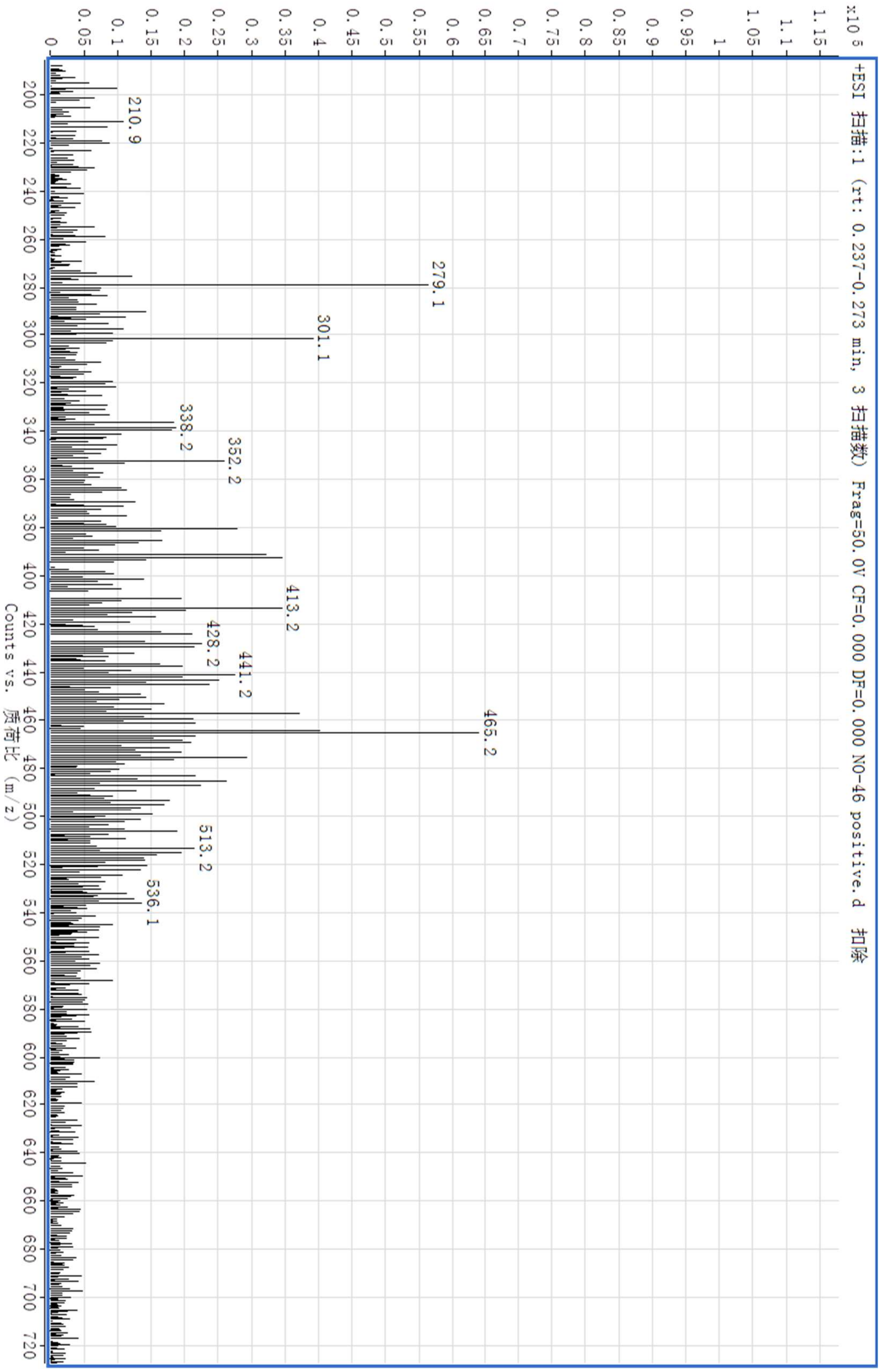

Figure S15. (+)-HRESIMS spectrum of **2**

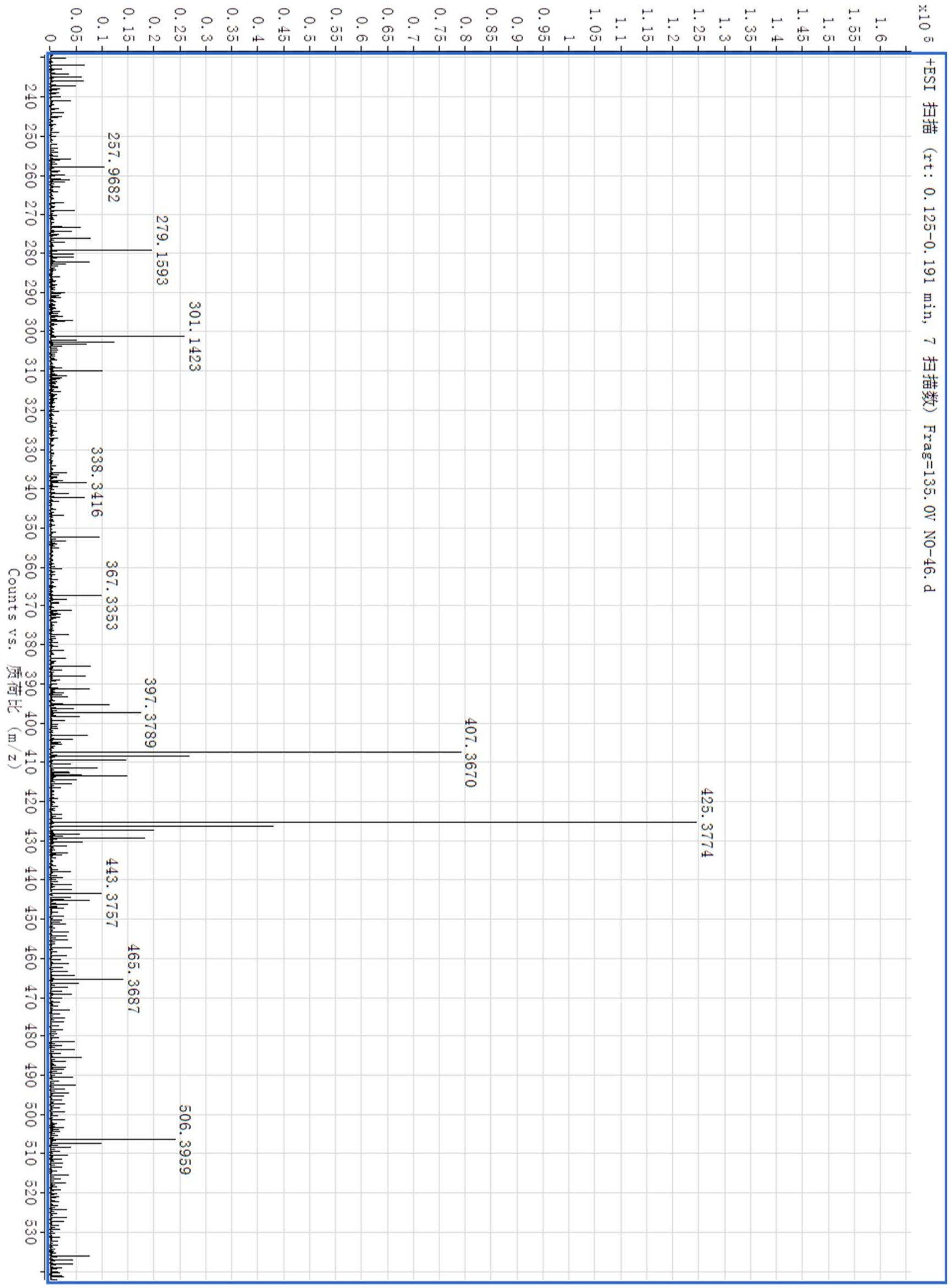

Figure S16. ^{1}H NMR spectrum of **3** in $CDCl_3$

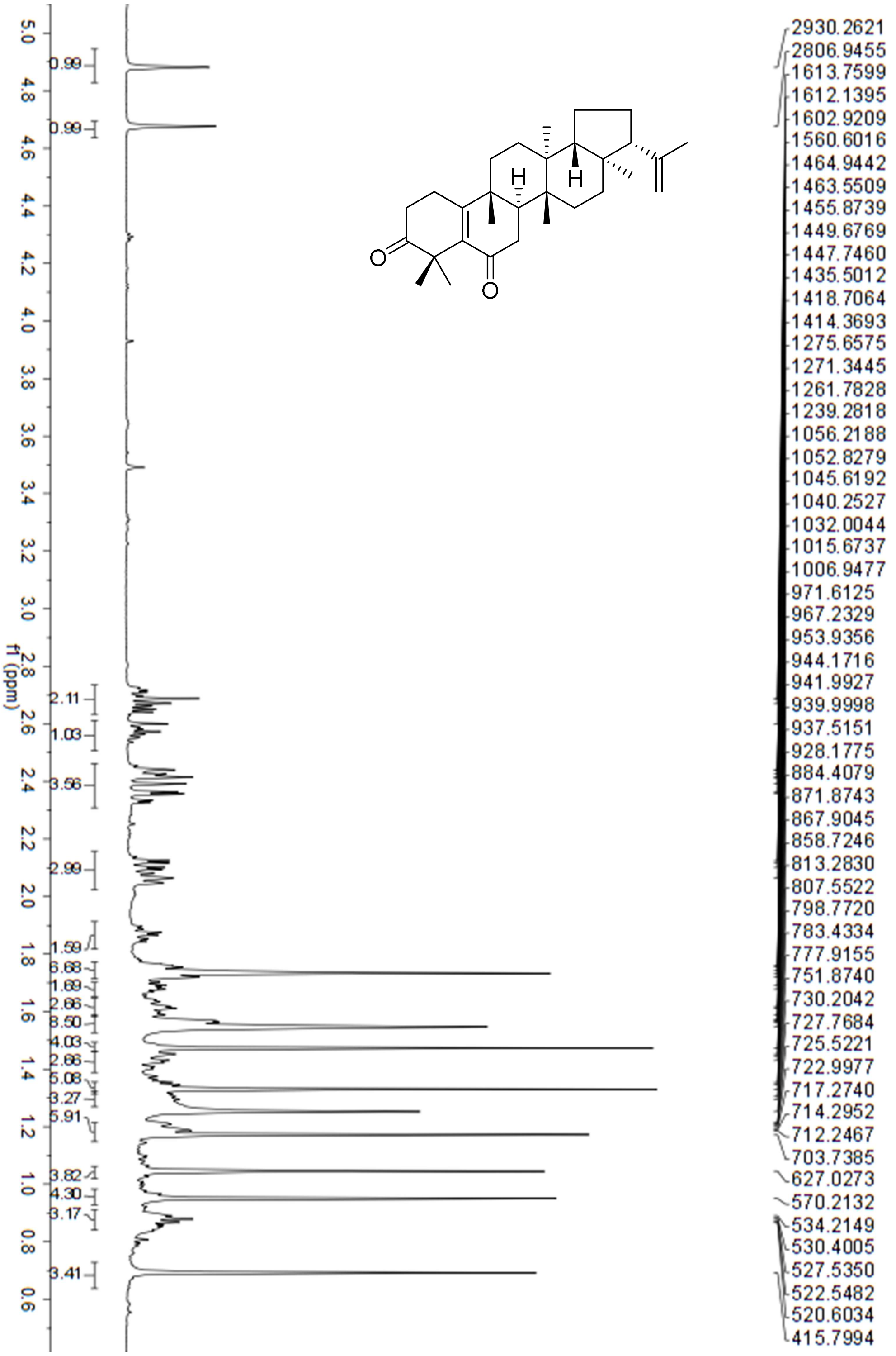

Figure S17. ^{13}C NMR spectrum of **3** in $CDCl_3$

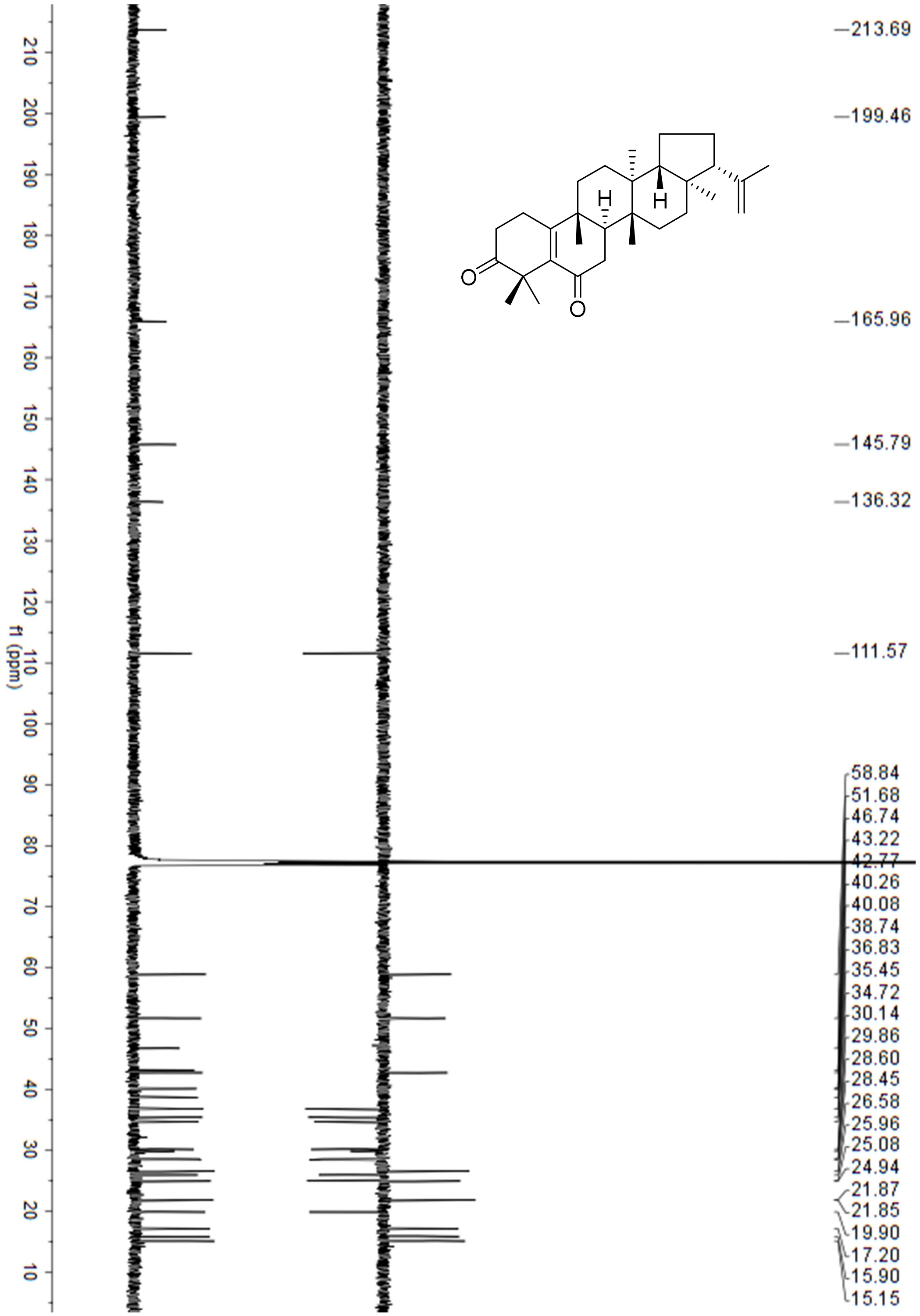

Figure S18. $^{1}H-^{1}H$ COSY NMR spectrum of **3** in $CDCl_3$

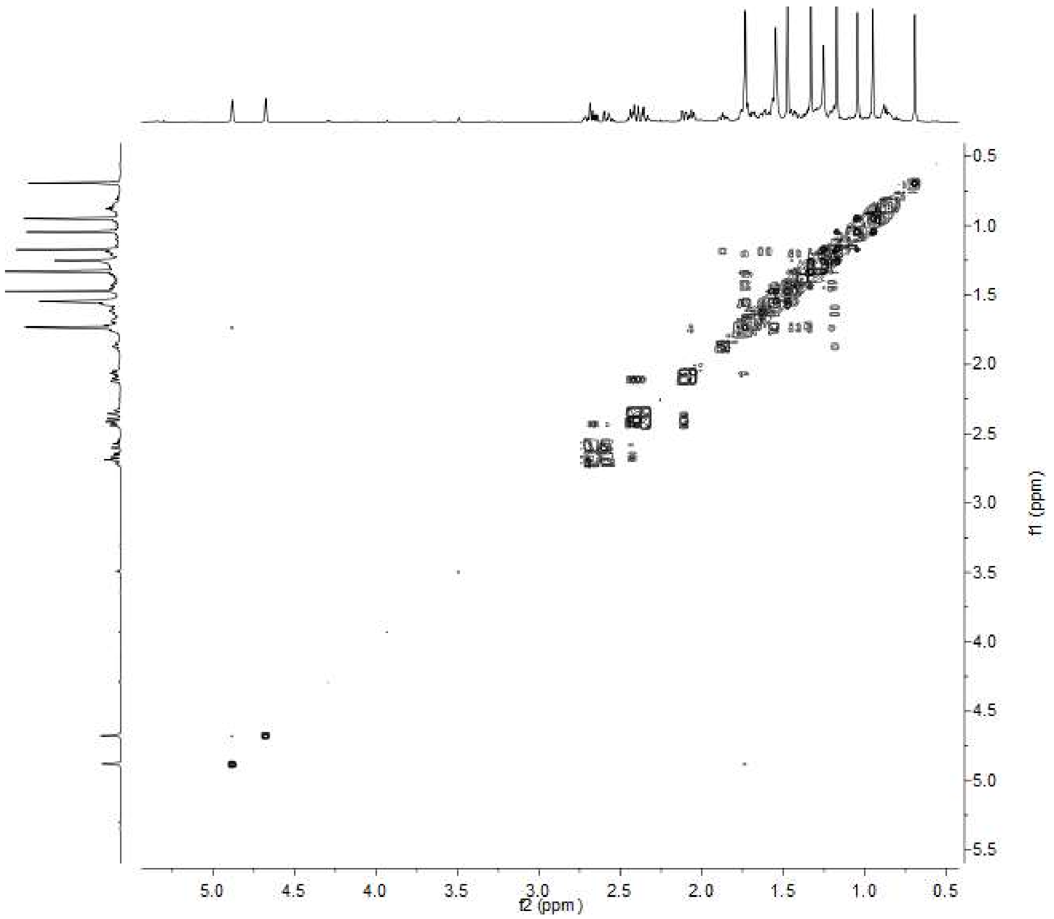

Figure S19. HSQC NMR spectrum of **3** in $CDCl_3$

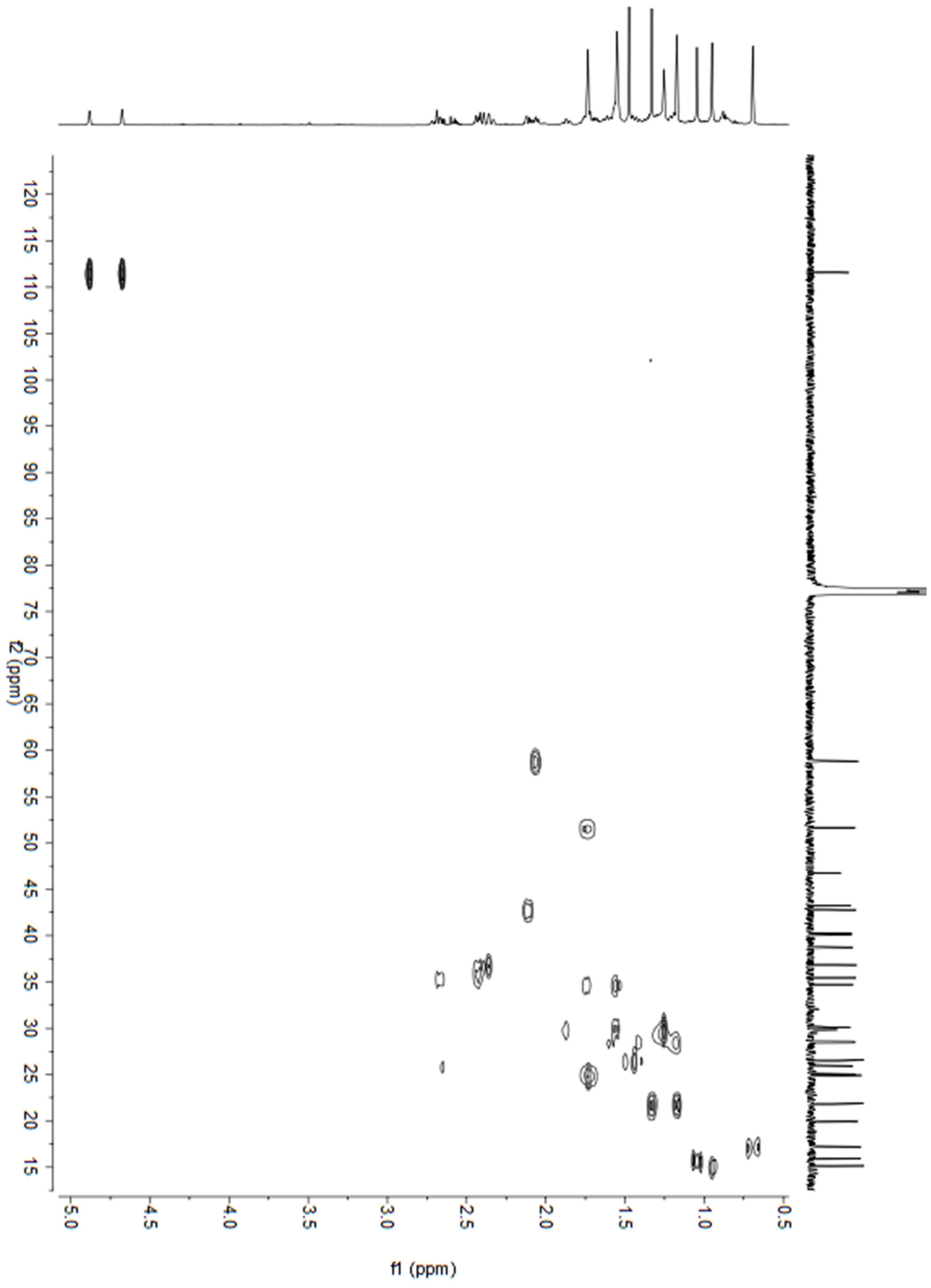

Figure S20. HMBC NMR spectrum of **3** in $CDCl_3$

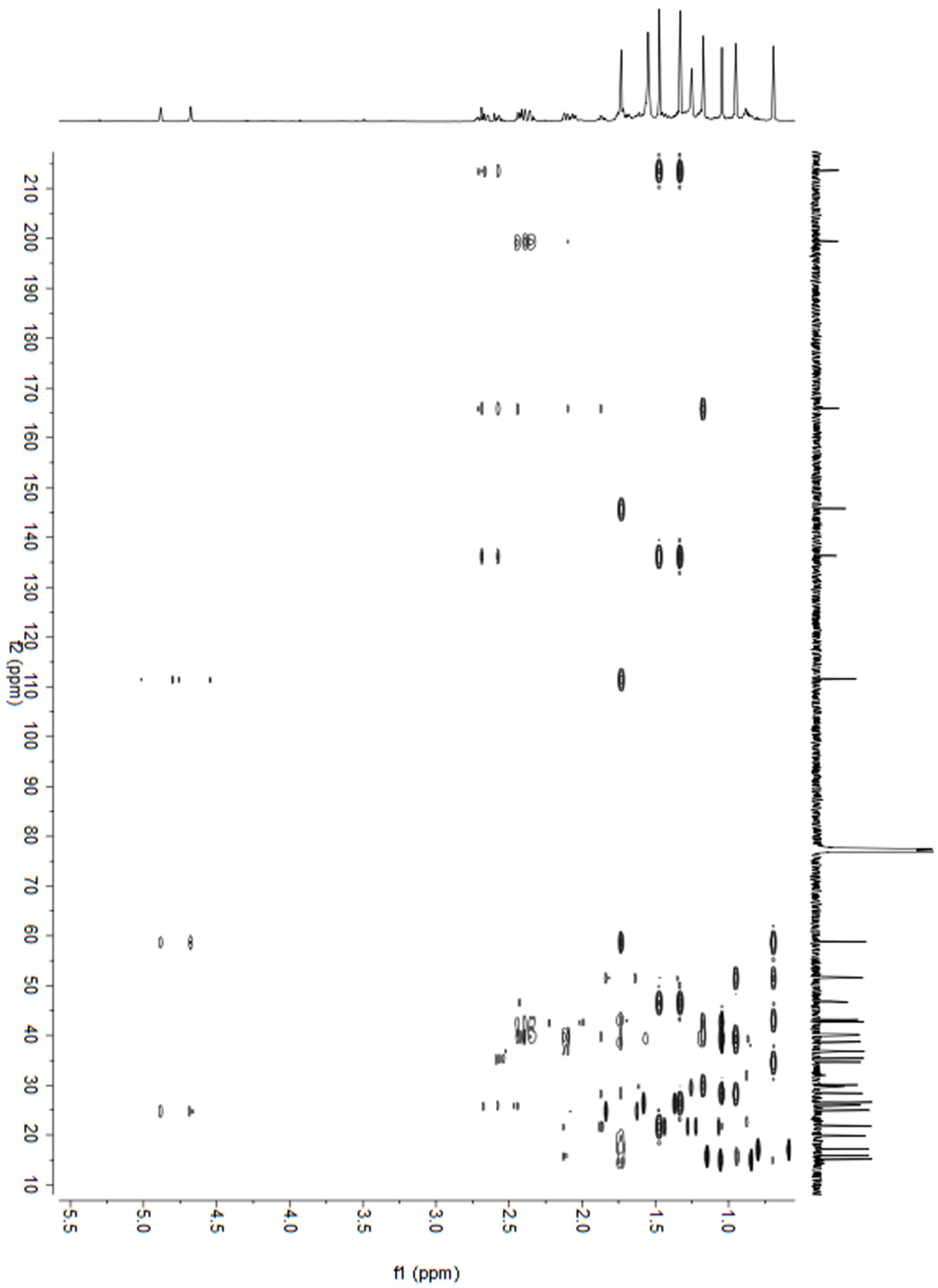

Figure S21. ROESY NMR spectrum of **3** in $CDCl_3$

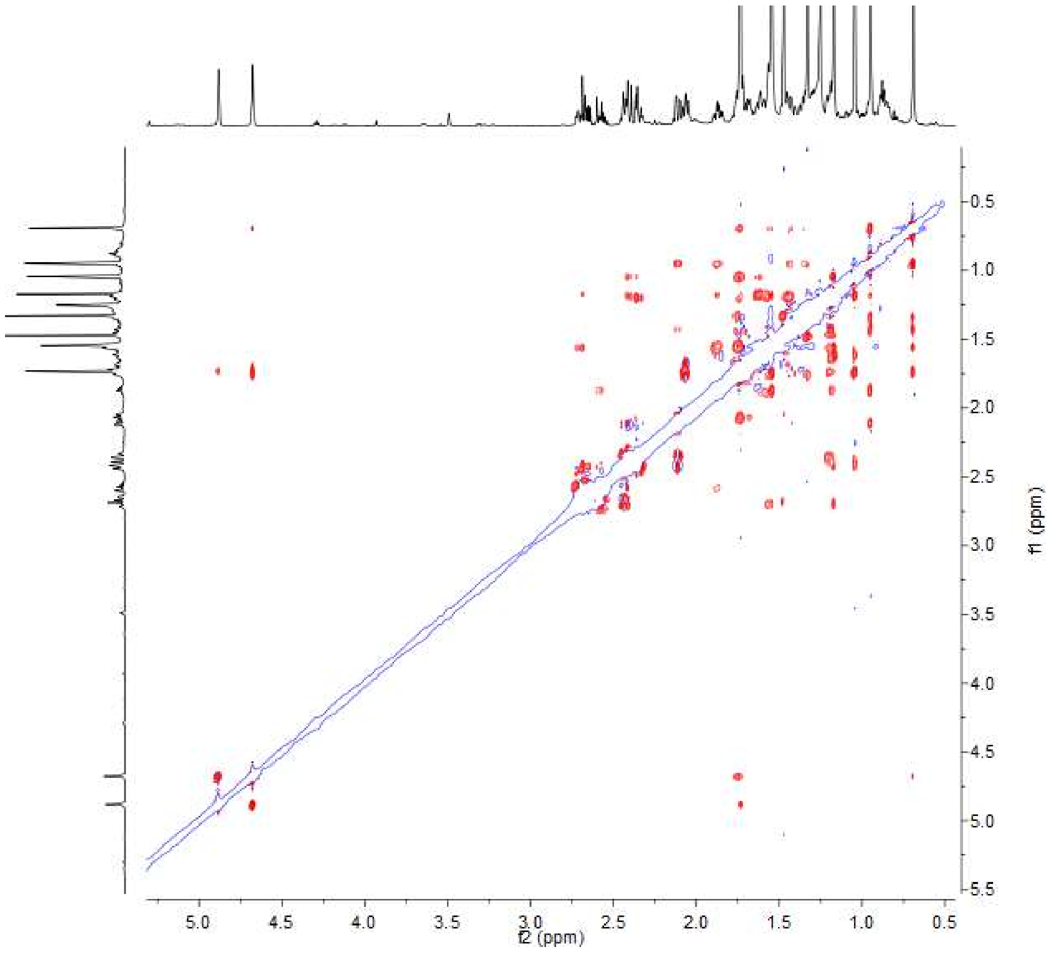

Figure S22. (+)-LRESIMS spectrum of **3**

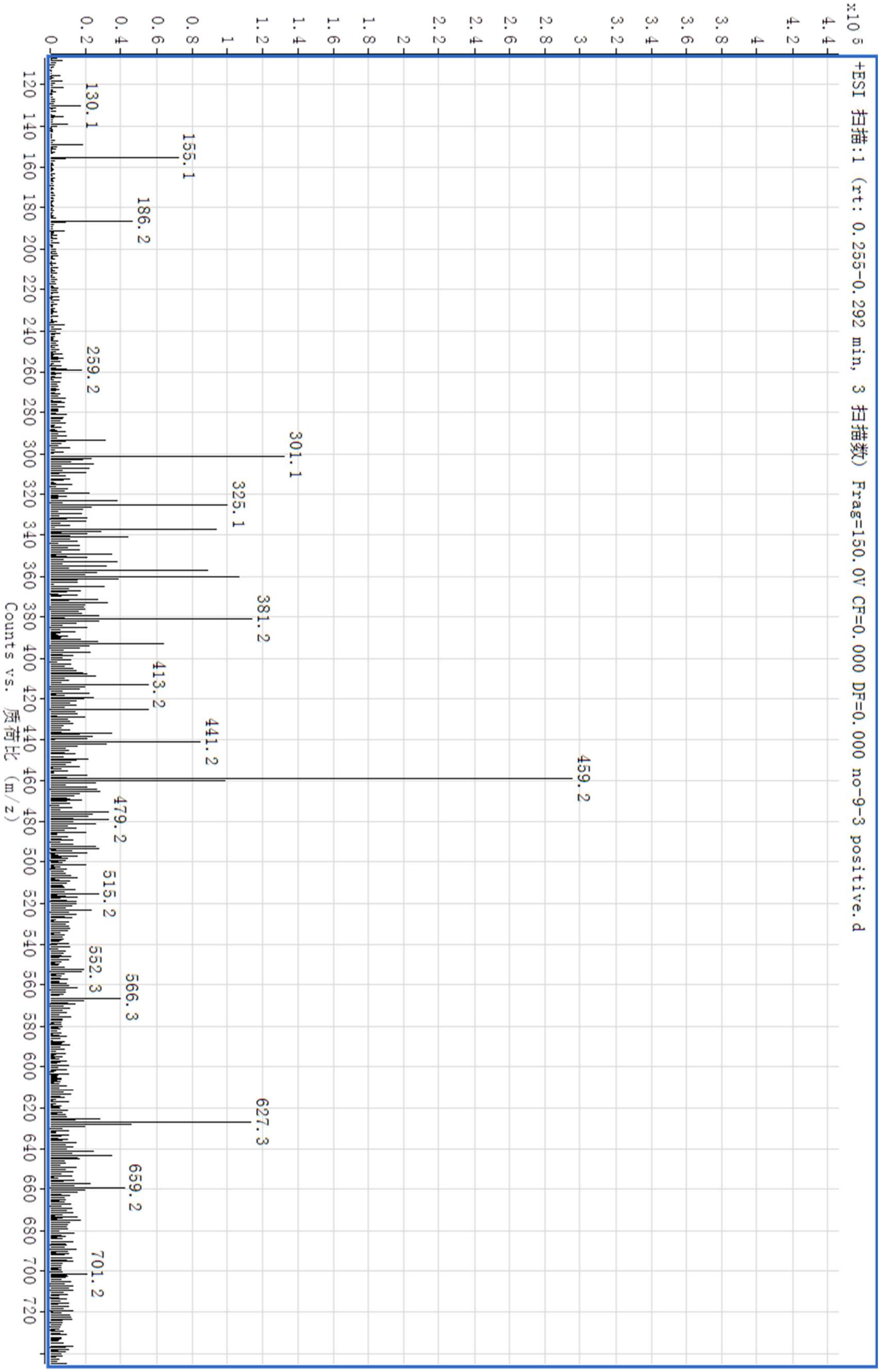

Figure S23. (+)-HRESIMS spectrum of **3**

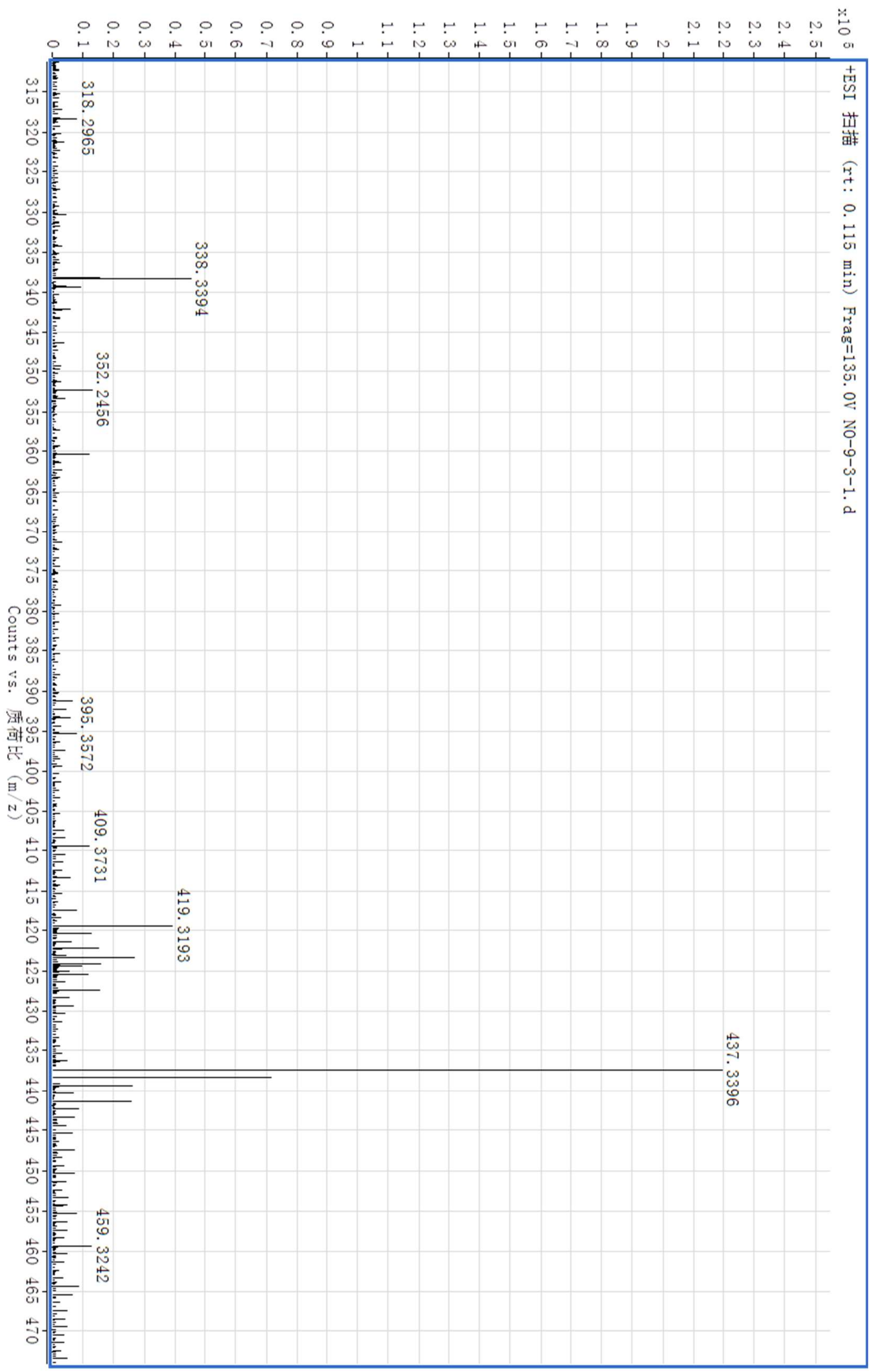

Figure S24. UV spectrum of **3**

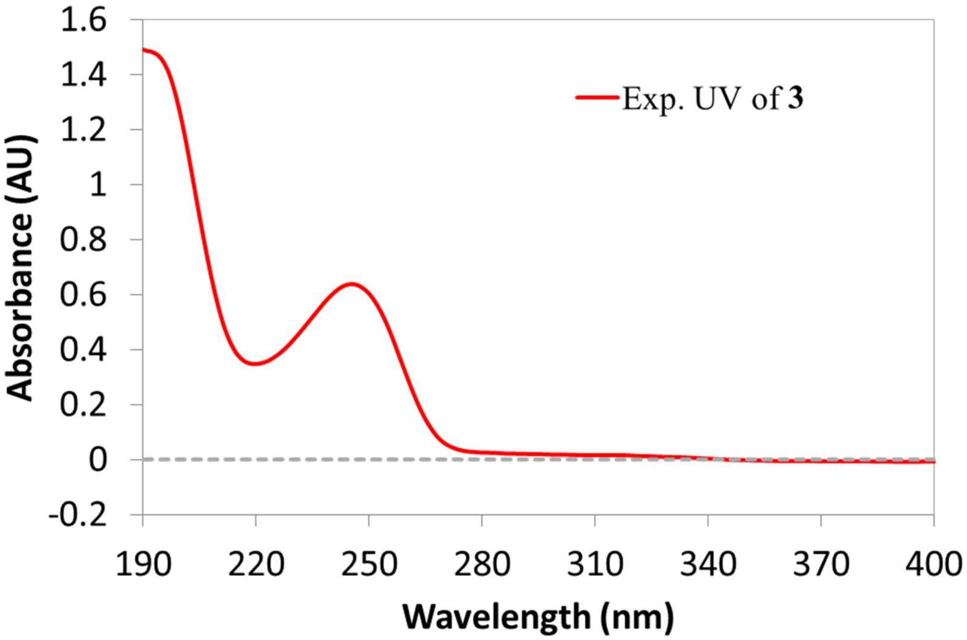

Figure S25. ^{1}H NMR spectrum of **4** in $CDCl_3$

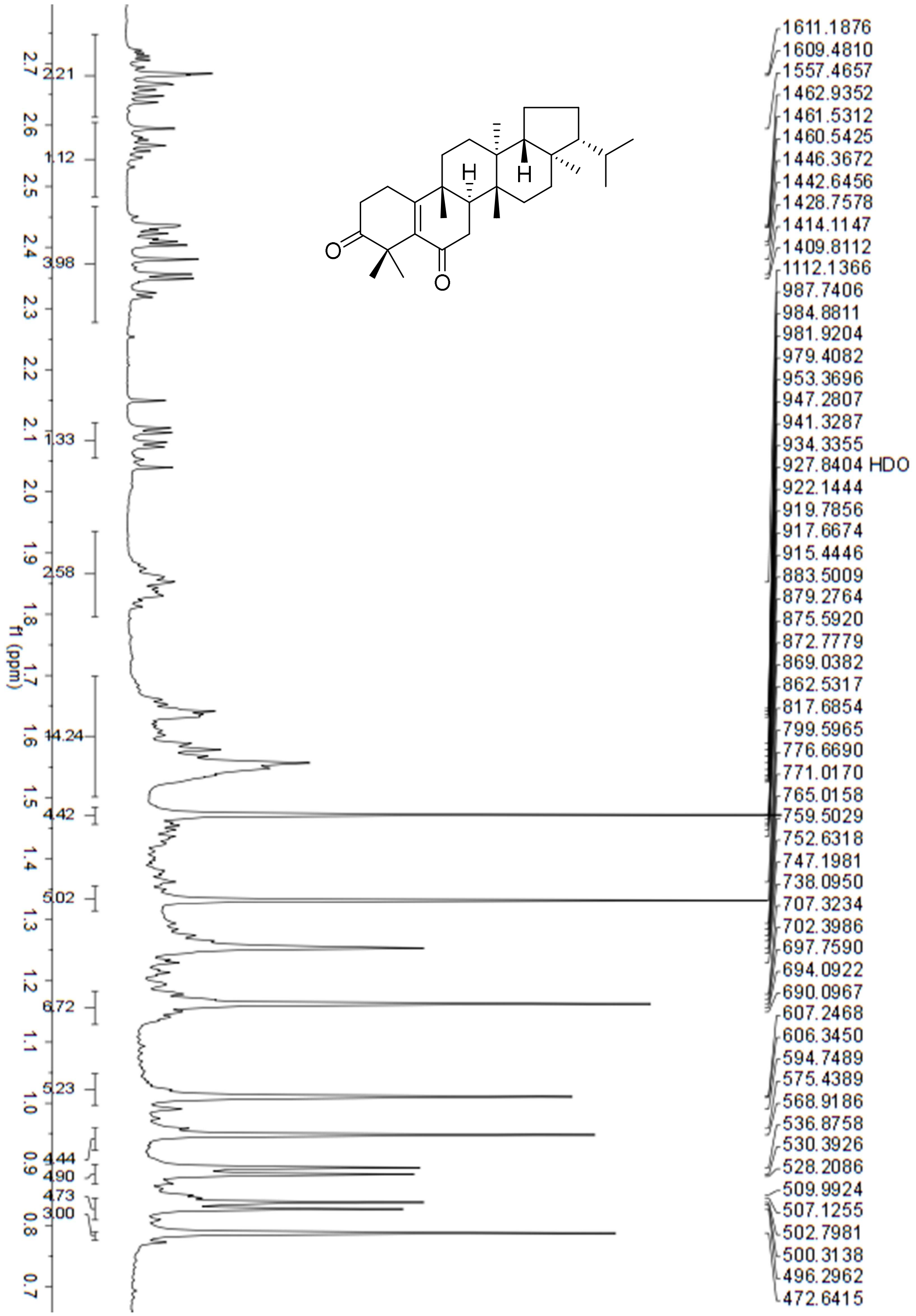

Figure S26. ^{13}C NMR spectrum of **4** in $CDCl_3$

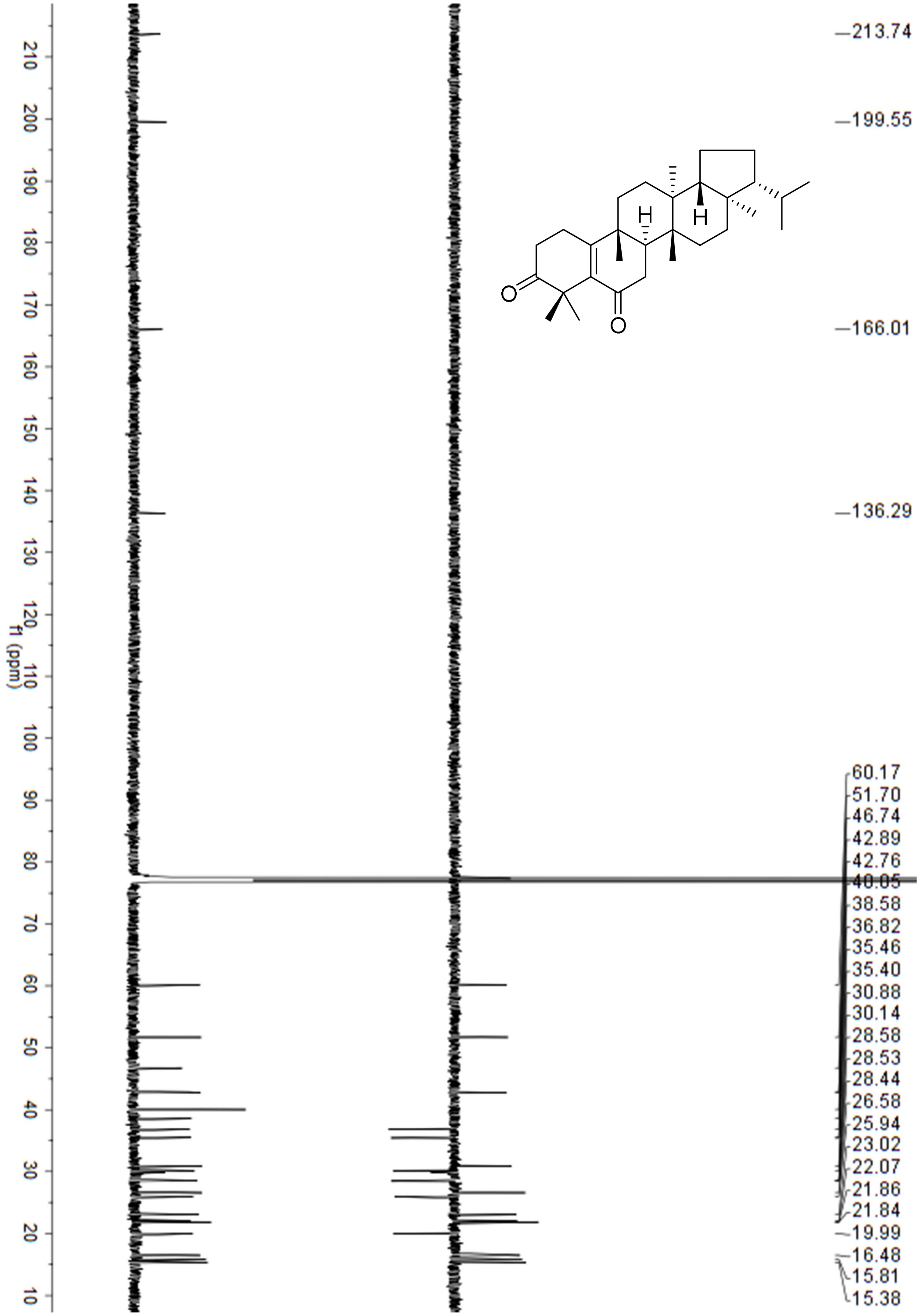

Figure S27. ^1H–^1H COSY NMR spectrum of **4** in $CDCl_3$

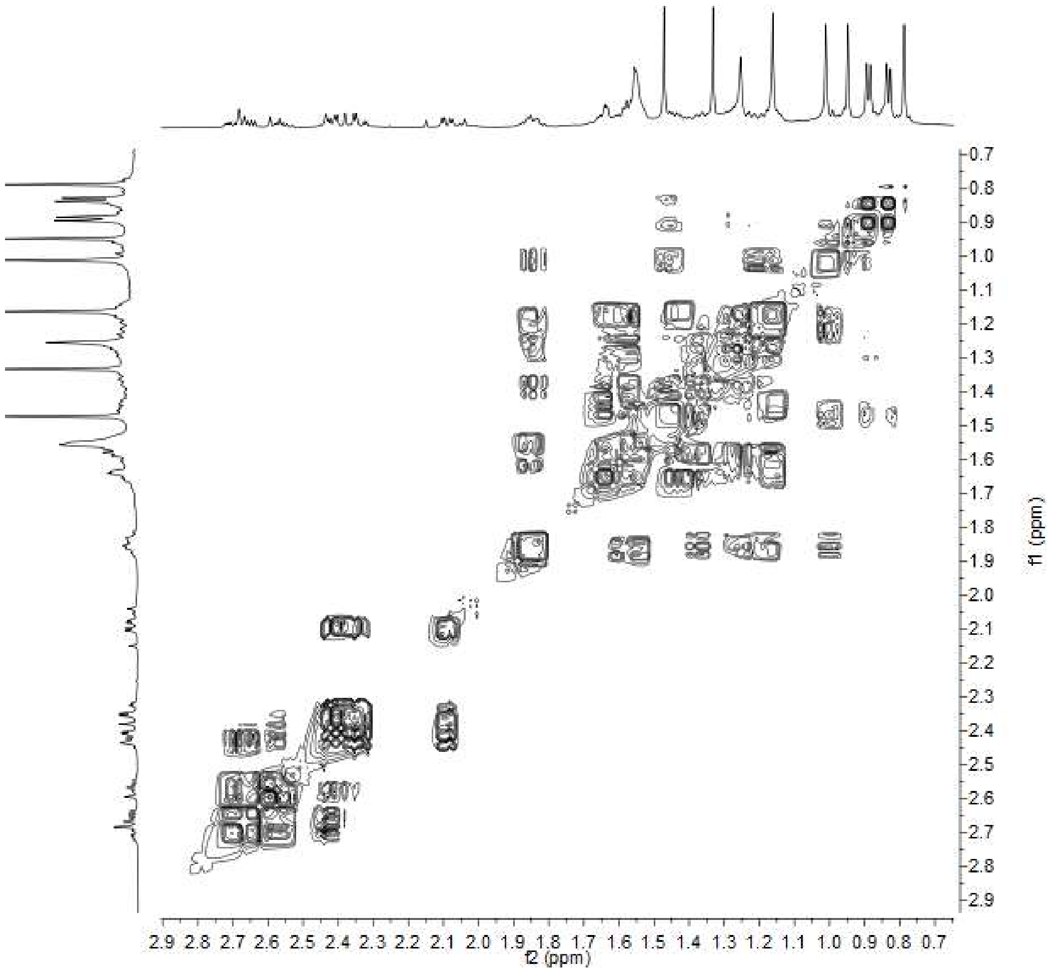

Figure S28. HSQC NMR spectrum of **4** in $CDCl_3$

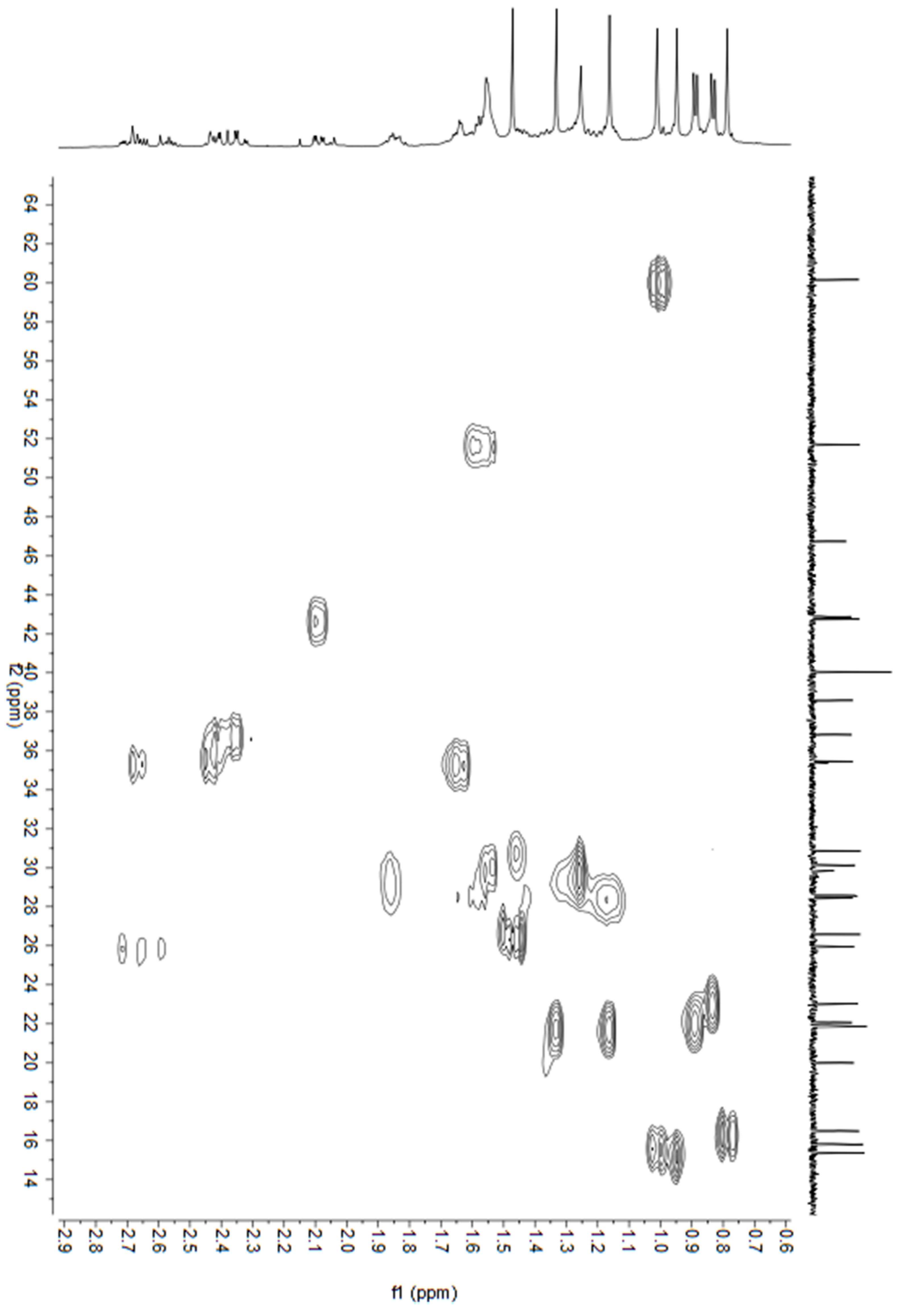

Figure S29. HMBC NMR spectrum of **4** in $CDCl_3$

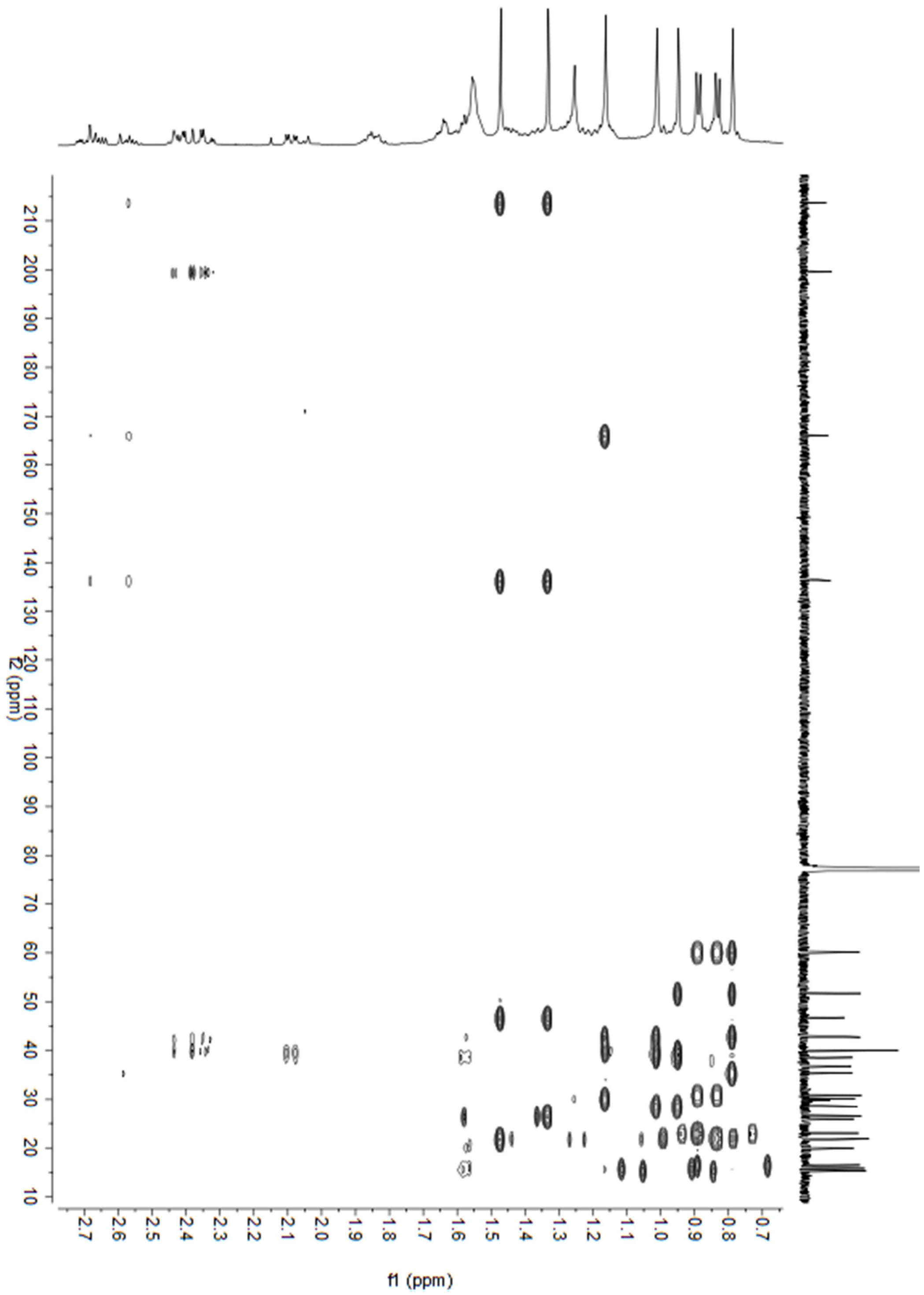

Figure S30. ROESY NMR spectrum of **4** in $CDCl_3$

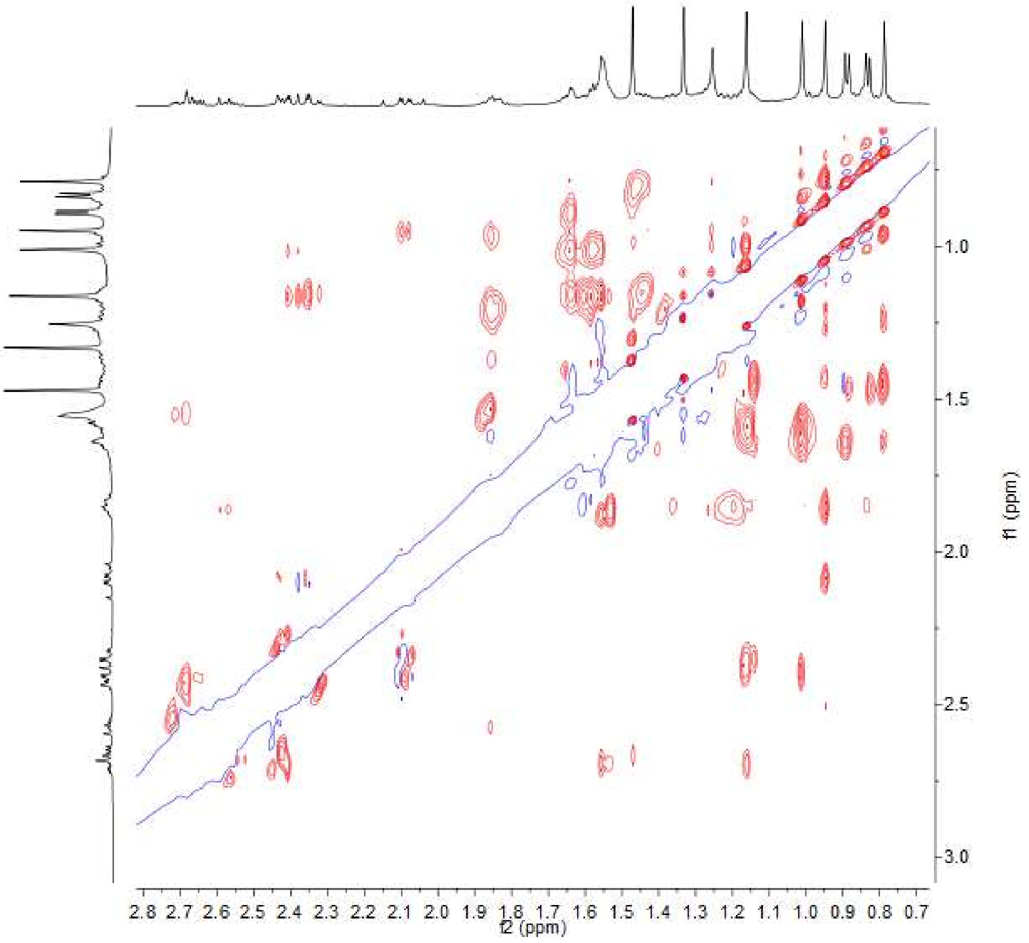

Figure S31. (+)-LRESIMS spectrum of **4**

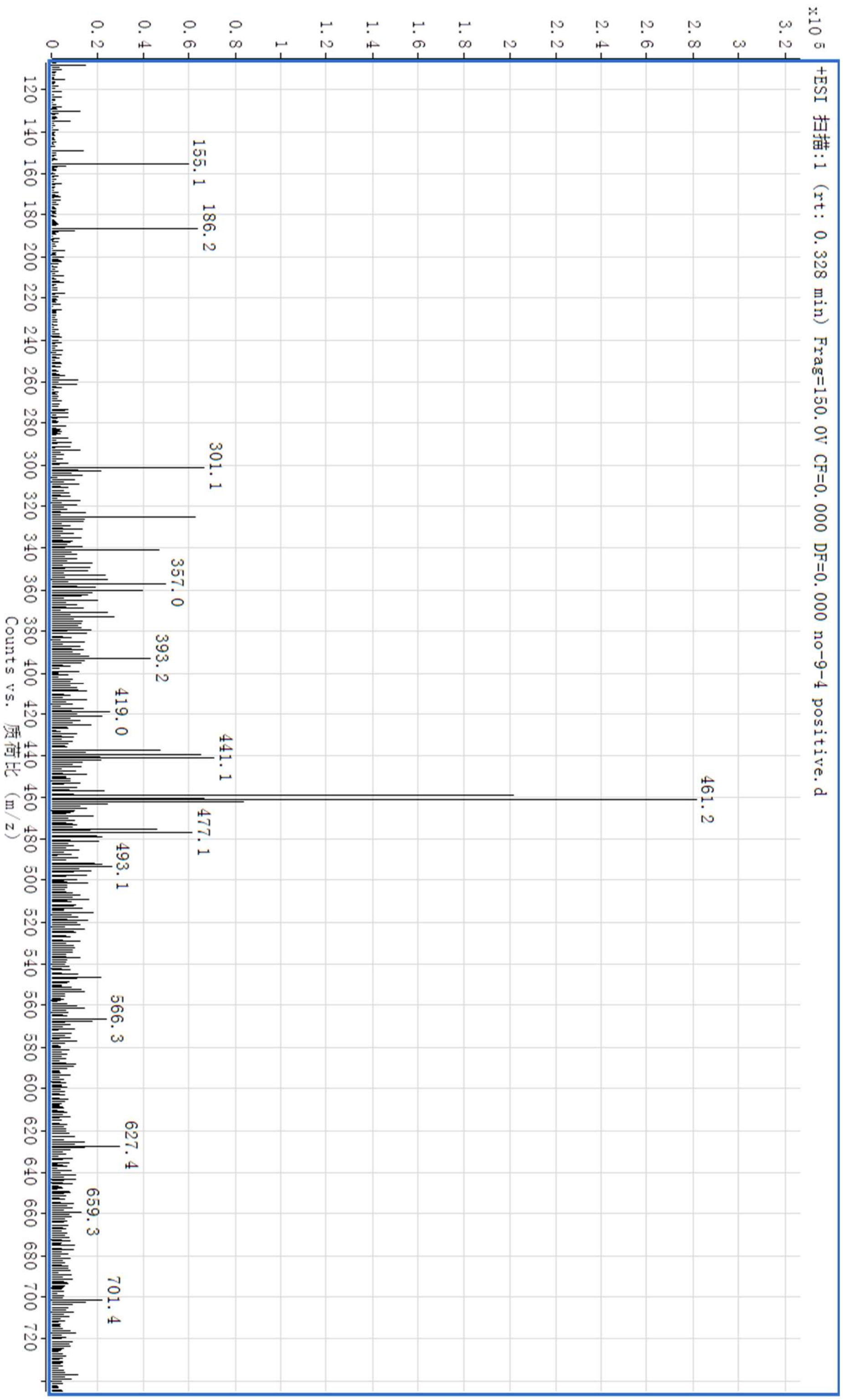

Figure S32. (+)-HRESIMS spectrum of **4**

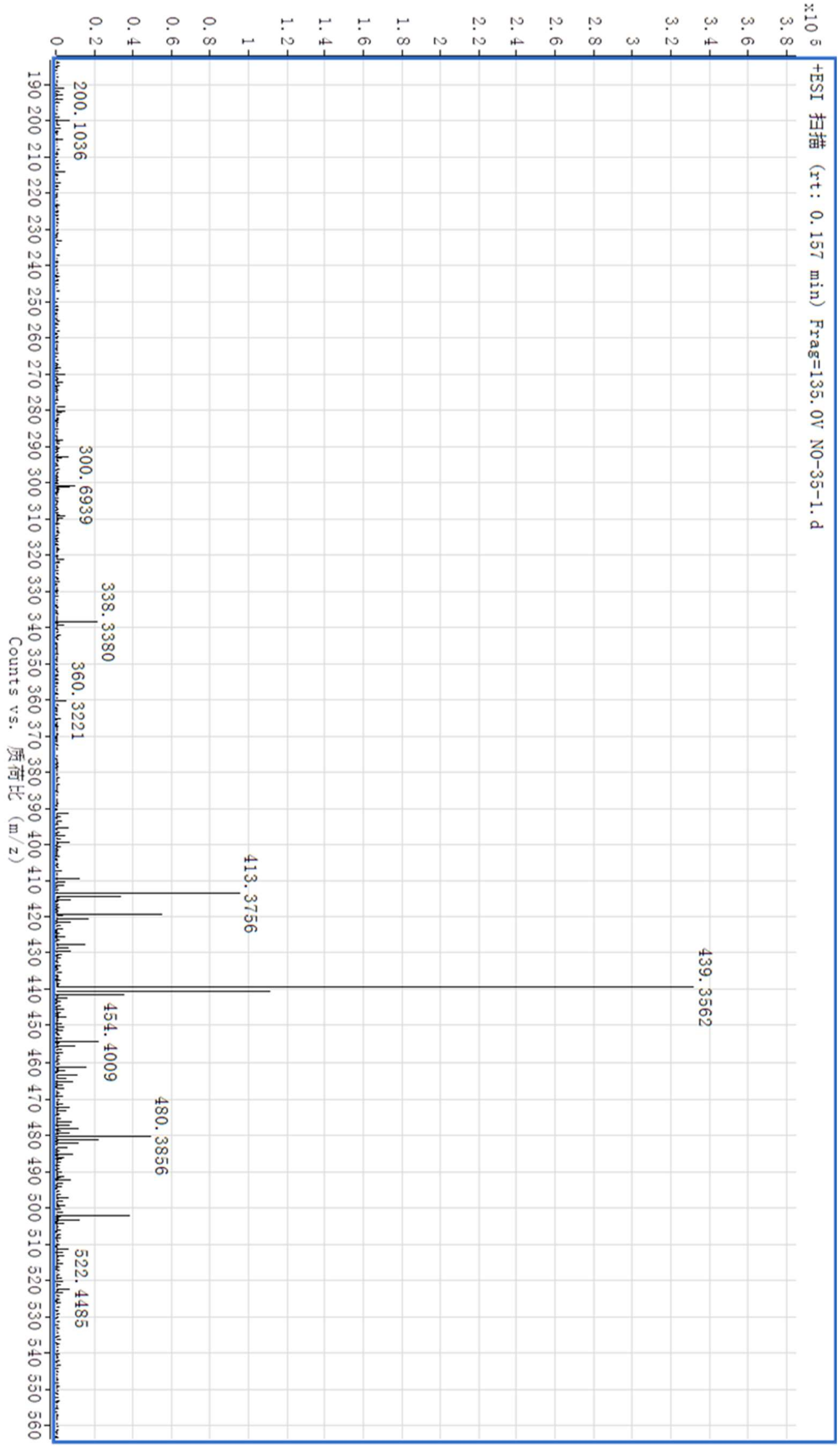

Figure S33. UV spectrum of **4**

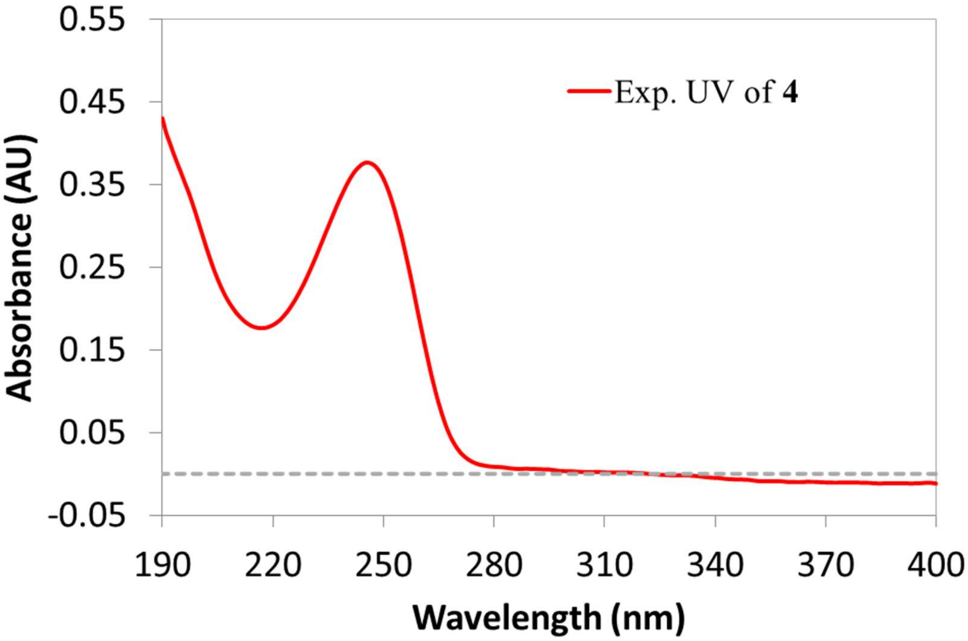

Figure S34. ^{1}H NMR spectrum of **5** in C_5D_5N

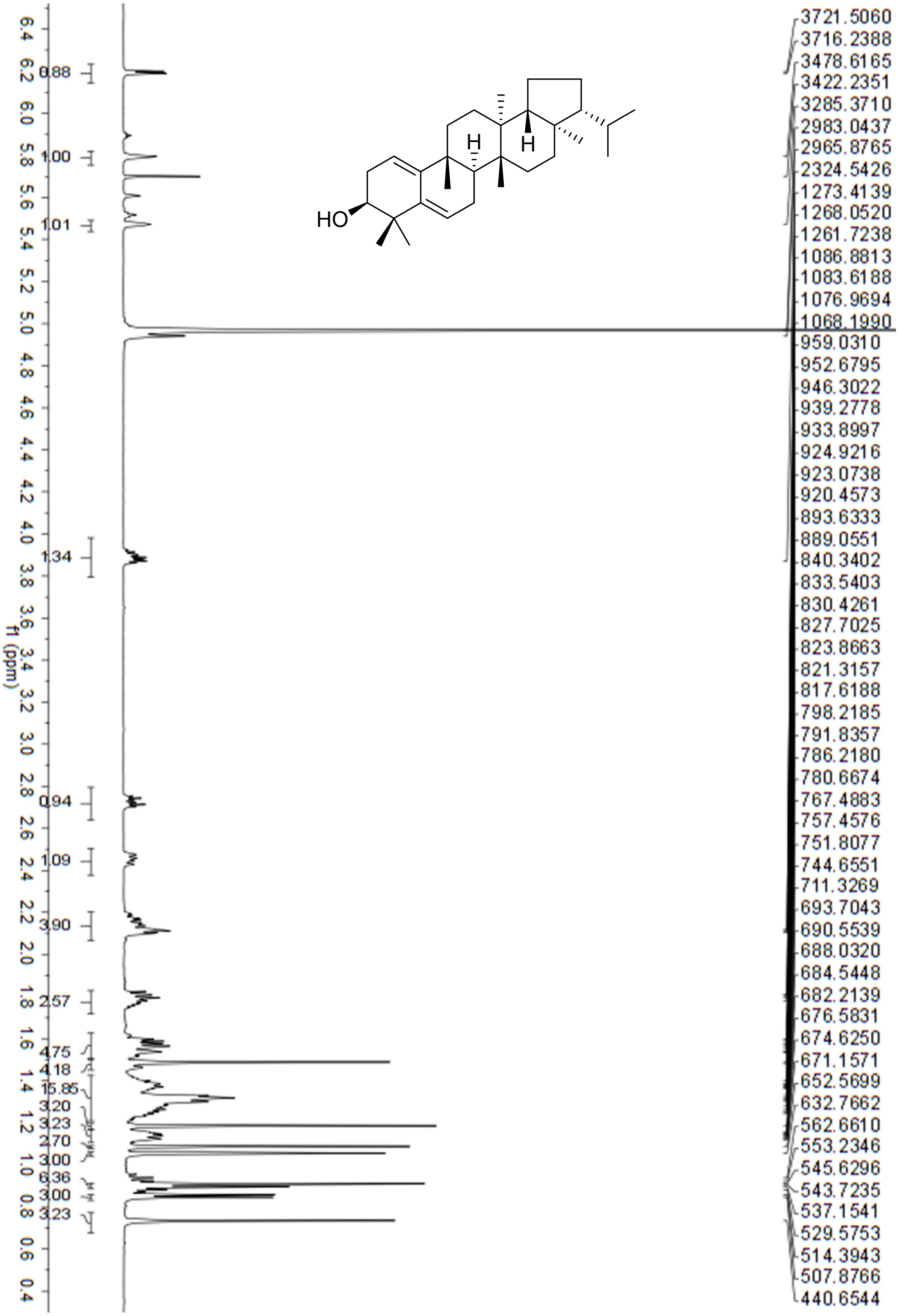

Figure S35. ^{13}C NMR spectrum of **5** in C_5D_5N

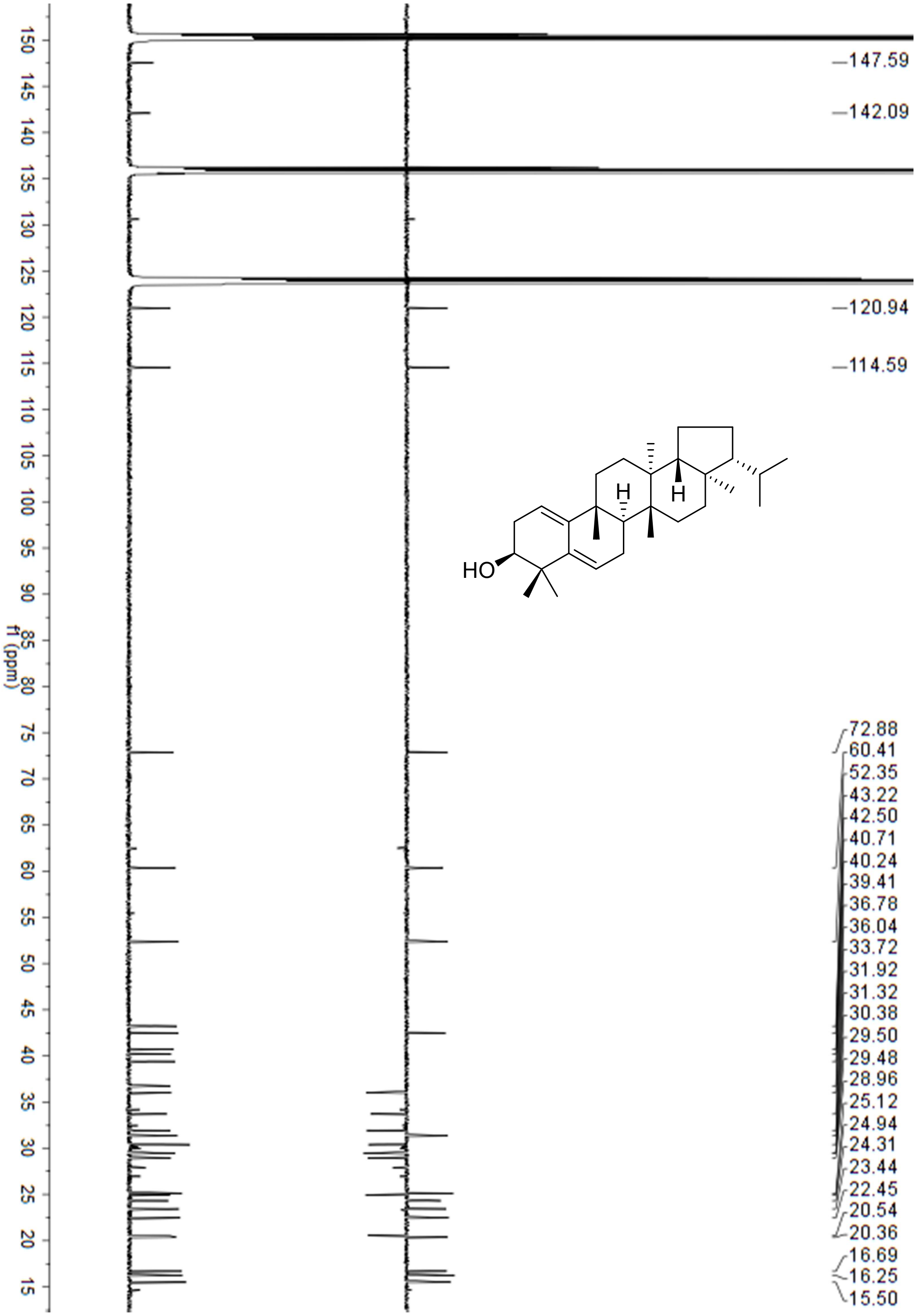

Figure S36. $^{1}H-^{1}H$ COSY NMR spectrum of **5** in C_5D_5N

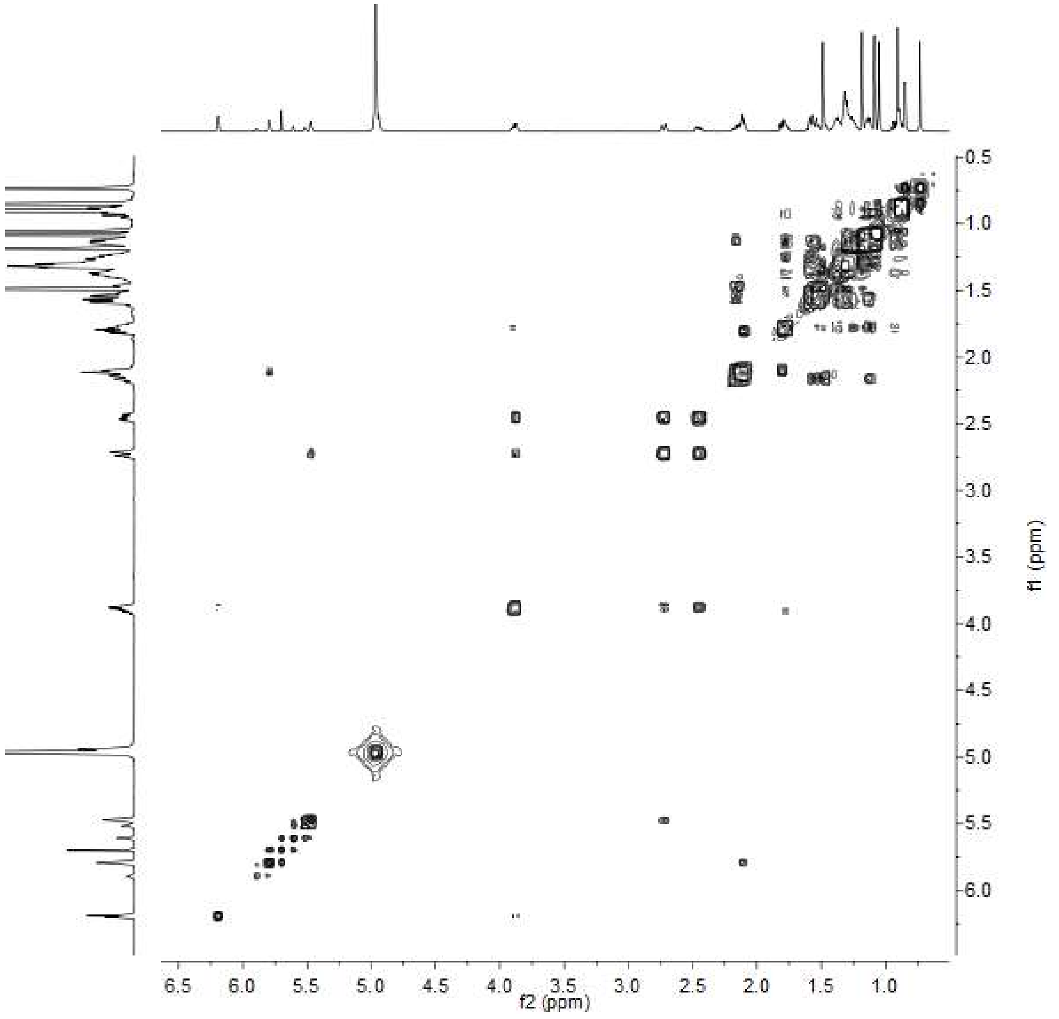

Figure S37. HSQC NMR spectrum of **5** in C_5D_5N

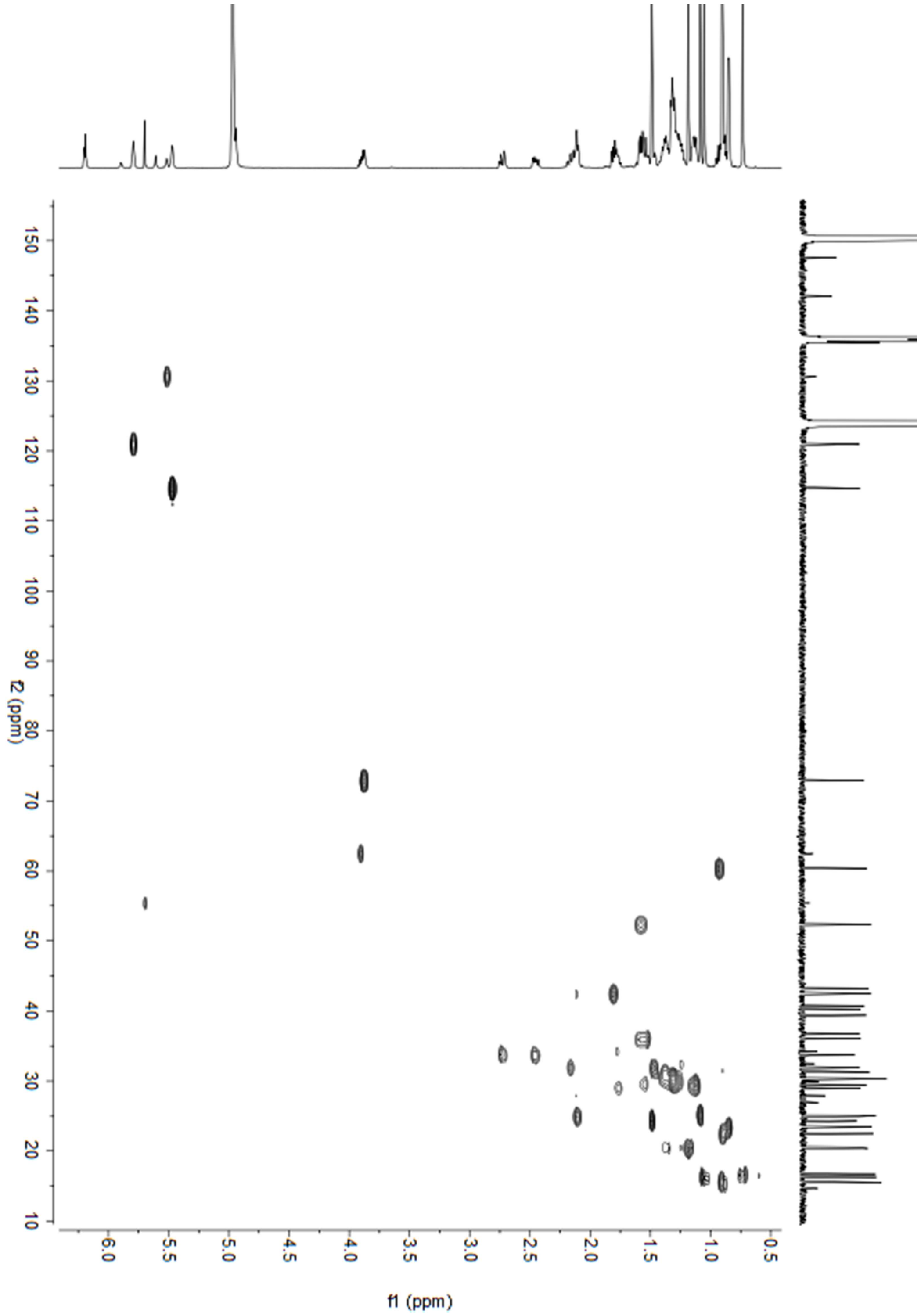

Figure S38. HMBC NMR spectrum of **5** in C_5D_5N

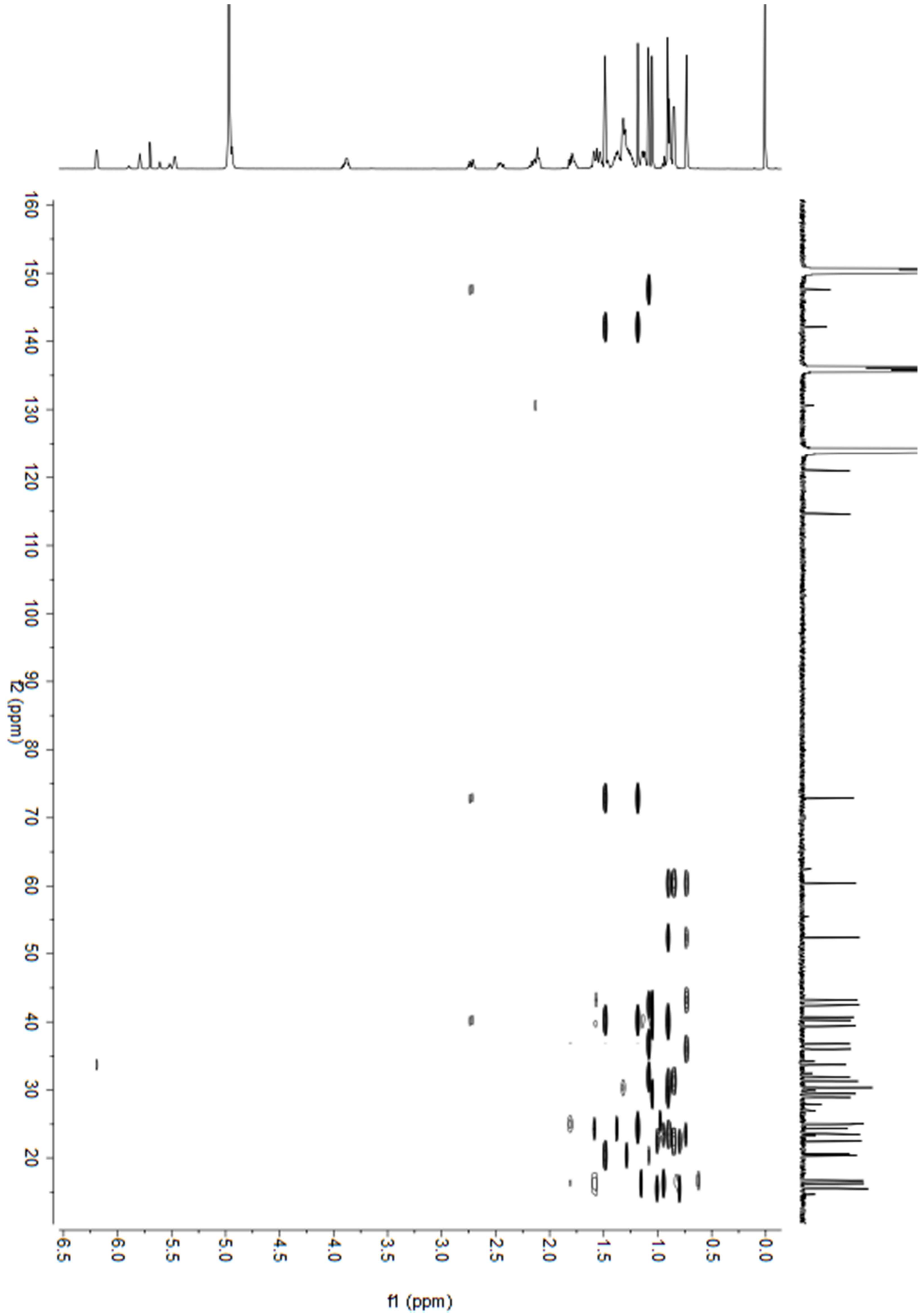

Figure S39. ROESY NMR spectrum of **5** in C_5D_5N

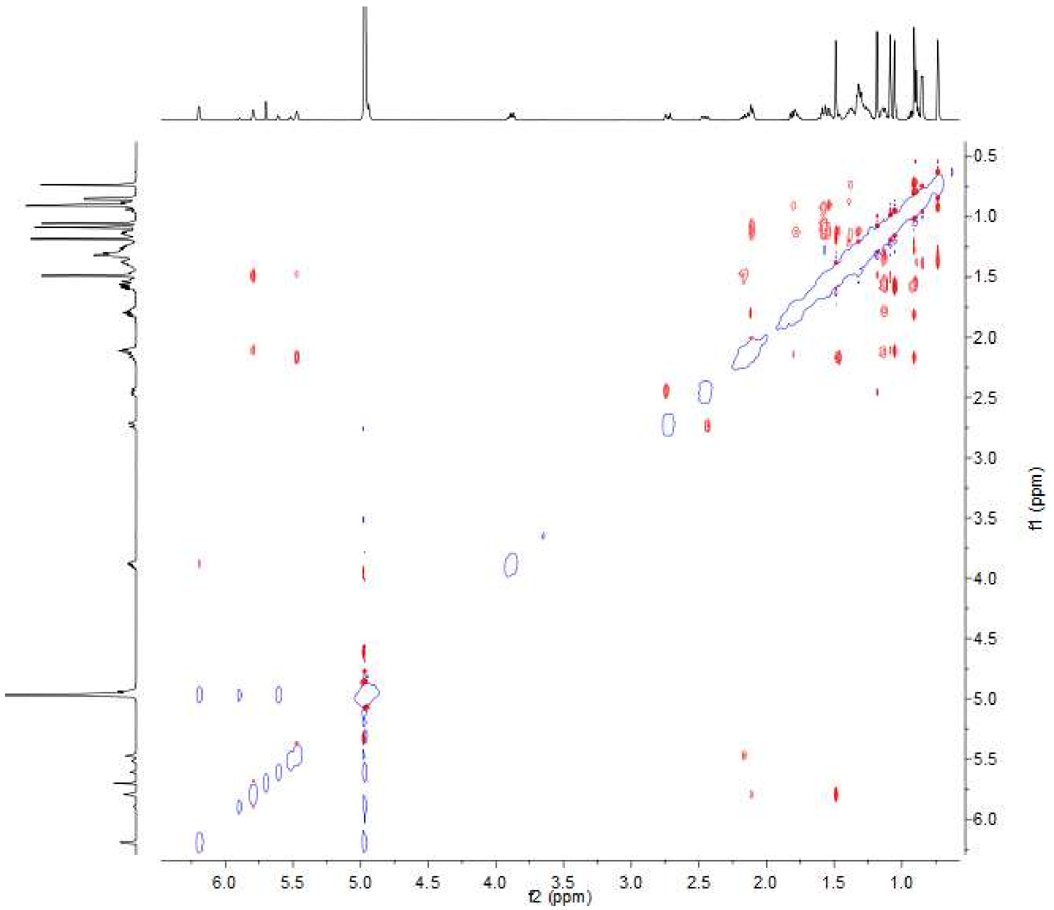

Figure S40. (+)-LRESIMS spectrum of **5**

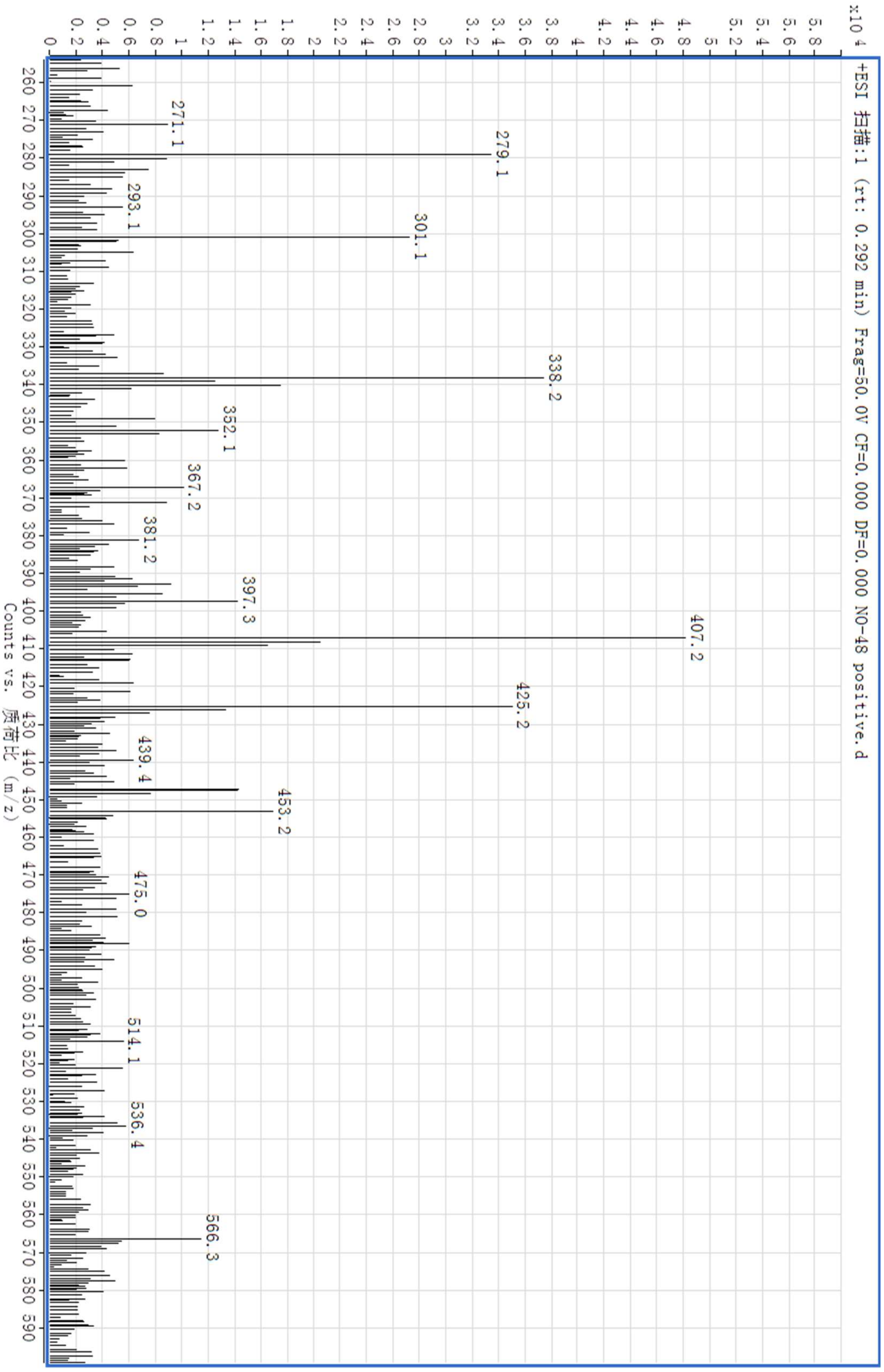

Figure S41. (+)-HRESIMS spectrum of **5**

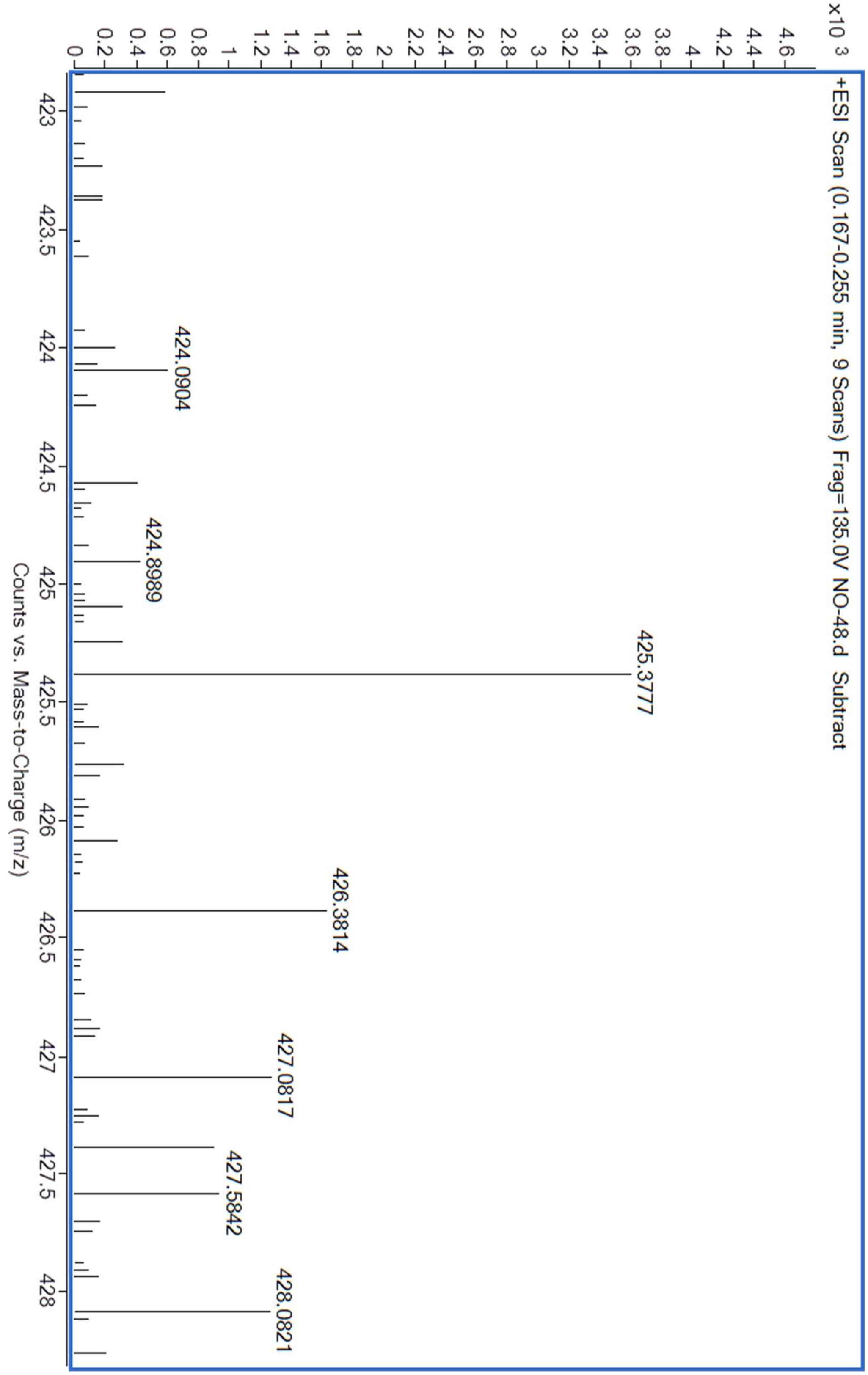

Figure S42. UV spectrum of **5**

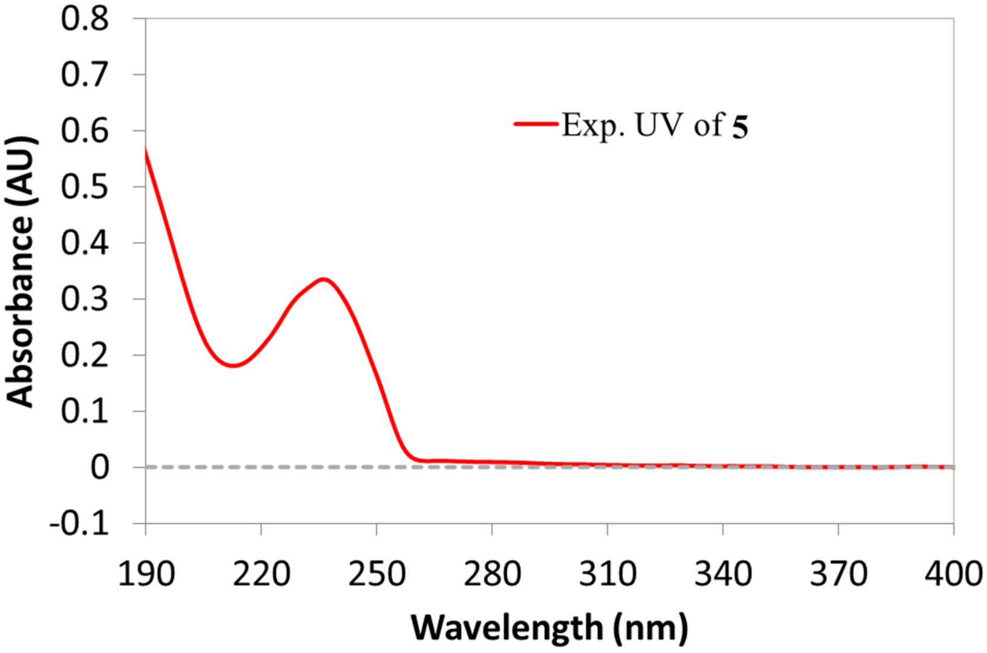

Figure S43. The HPLC analysis for the purity of compound **1**

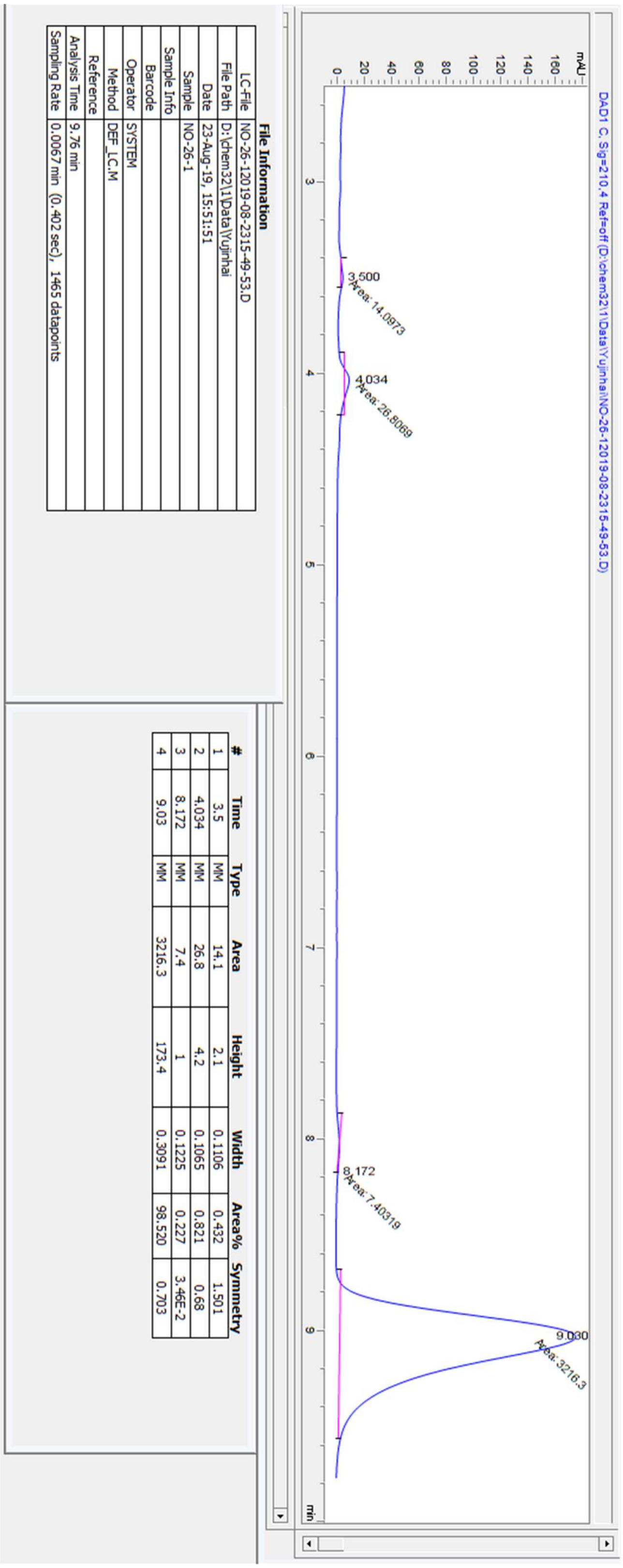

Figure S44. The HPLC analysis for the purity of compound **2**

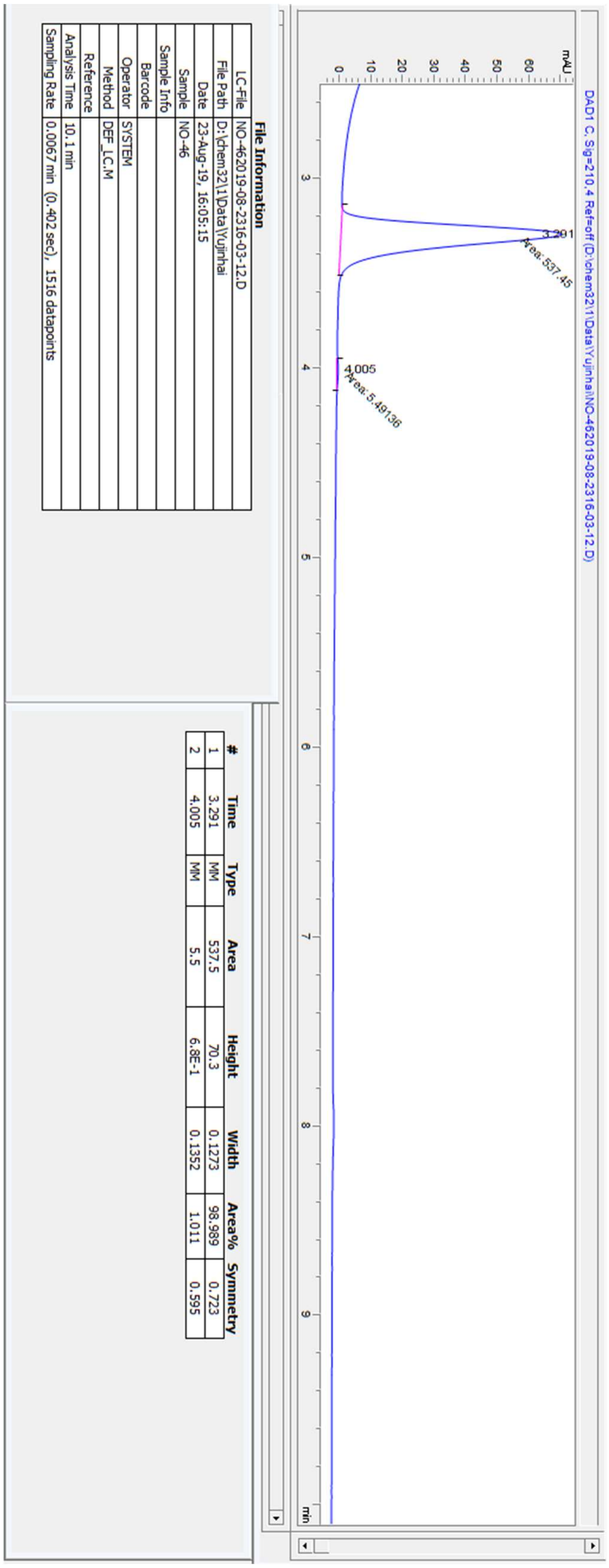

Figure S45. The HPLC analysis for the purity of compound **3**

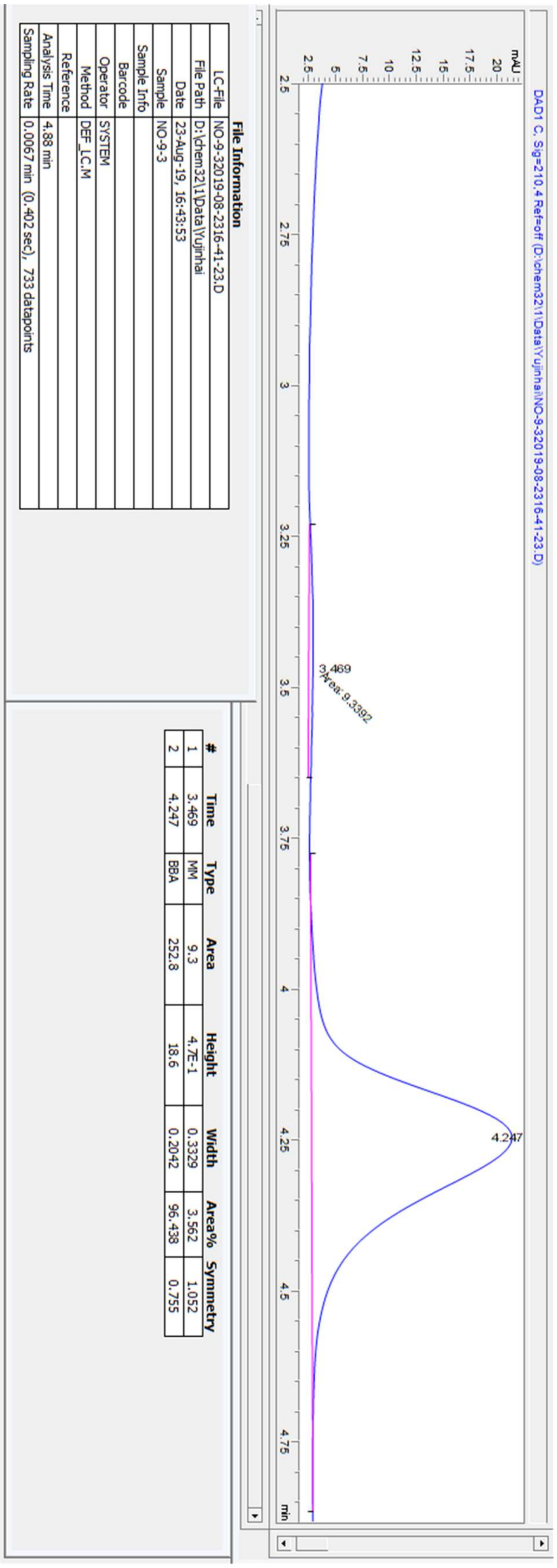

Figure S46. The HPLC analysis for the purity of compound **4**

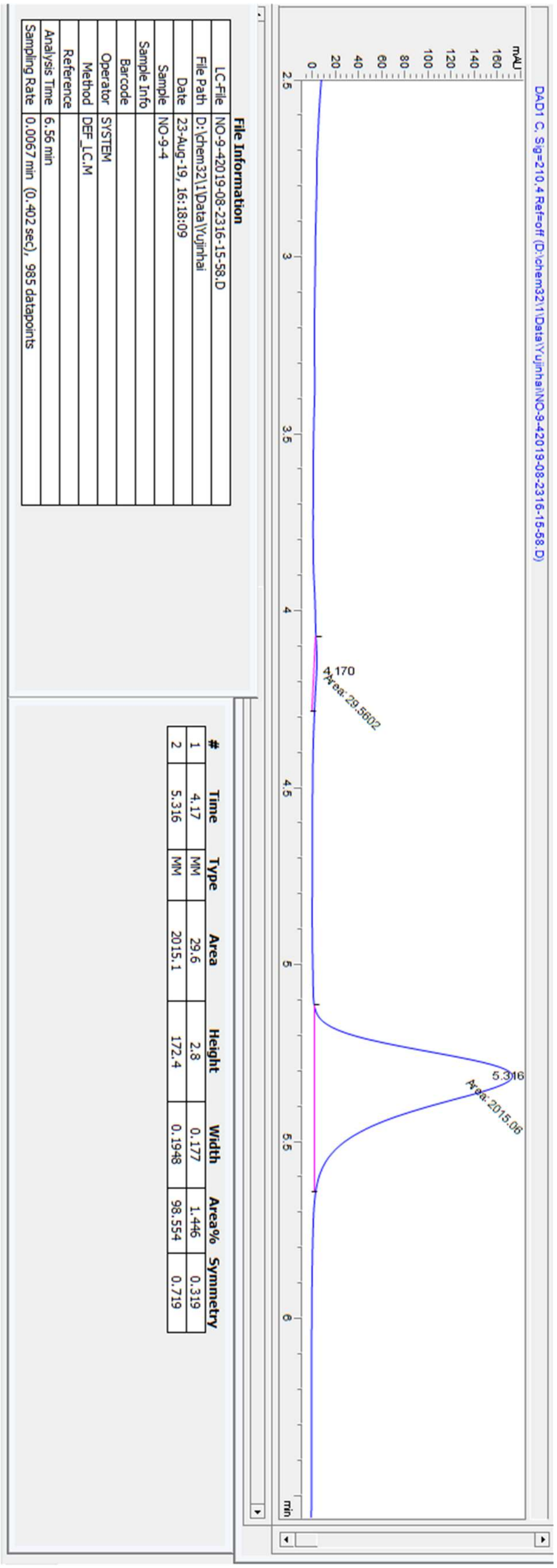

Figure S47. The HPLC analysis for the purity of compound **5**

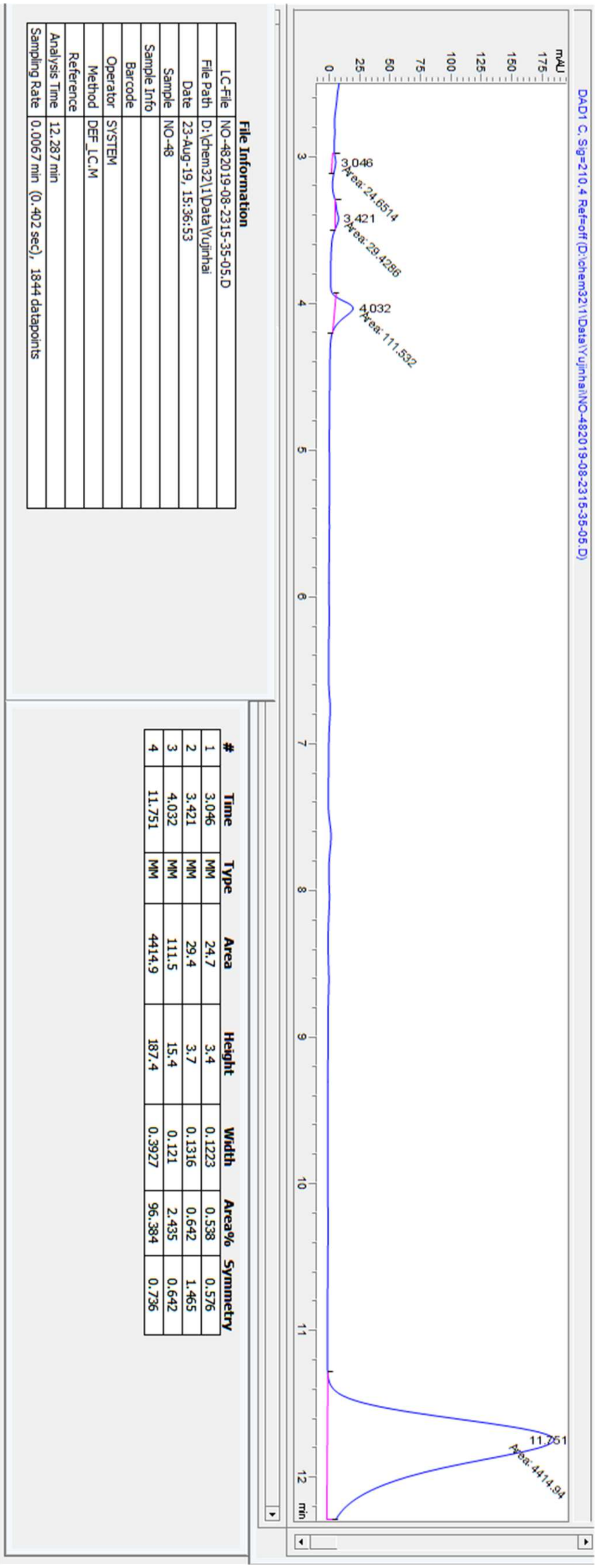

File Information

LC-File	NO-482019-08-2315-35-05.D
File Path	D:\chem32\1\Data\Yujinhai
Date	23-Aug-19, 15:36:53
Sample	NO-48
Sample Info	
Barcode	
Operator	SYSTEM
Method	DEF_LC.M
Reference	
Analysis Time	12.287 min
Sampling Rate	0.0067 min (0.402 sec), 1844 datapoints

#	Time	Type	Area	Height	Width	Area%	Symmetry
1	3.046	MM	24.7	3.4	0.1223	0.538	0.576
2	3.421	MM	29.4	3.7	0.1316	0.642	1.465
3	4.032	MM	111.5	15.4	0.121	2.435	0.642
4	11.751	MM	4414.9	187.4	0.3927	96.384	0.736
